# A Comprehensive Review of the Pharmacology, Chemistry, Traditional Uses and Quality Control of Star Anise (*Illicium verum* Hook. F.): An Aromatic Medicinal Plant

**DOI:** 10.3390/molecules28217378

**Published:** 2023-11-01

**Authors:** Qiyuan Zou, Yuanyuan Huang, Wenyan Zhang, Chen Lu, Jingquan Yuan

**Affiliations:** 1 Scientific Experimental Center, Guangxi University of Chinese Medicine, 13 Wuhe Avenue, Nanning 530200, China; zouqiyuan1@163.com (Q.Z.); yuanyhuang@yeah.net (Y.H.); 2 Guangxi Botanical Garden of Medicinal Plants, Nanning 530023, China; 3 National Engineering Research Center for Southwest Endangered Medicinal Materials Resources Development, Nanning 530023, China

**Keywords:** *Illicium verum*, star anise, traditional uses, phytochemistry, pharmacology, toxicology, quality control

## Abstract

*Illicium verum* Hook. F., also known as star anise, is one of the most important plants of the genus Anise in the family Magnoliaceae. *I. verum* not only has the functions of warming Yang, dispersing cold, regulating Qi and relieving pain but can also be used as a condiment to increase flavor as well as reconcile and remove fish smells. Currently, 201 chemical constituents have been identified from star anise; among these, star anise oil and shikimic acid are the two most widely used and studied chemical components in star anise, with the oil accounting for a large proportion of the total. This review integrates, classifies and updates studies related to the botany, pharmacology, phytochemistry, traditional and modern uses and quality control of star anise, with a special reference to its phytochemical composition and pharmacological activity. It will provide a reference for further research on this important medicinal plant. In addition, the broad applications and research profiles of star anise essential oil and shikimic acid are highlighted. Our review indicates that the research prospects regarding star anise are very broad and worthy of further investigation.

## 1. Introduction

Plant-based Chinese medicine is a generic term for Chinese medicines derived from plant resources, which are processed from natural plants or parts of naturally occurring flora under the guidance of the Traditional Chinese Medicine (TCM) doctrine. The medicinal parts of plants for Chinese medicines include roots, rhizomes, stems, leaves, flowers, fruits, seeds, skins and whole herbs [1]. Before the rapid development of medicinal chemistry in the 20th century, human beings fought diseases with plants and other natural medicines. Even in the rapidly developing 21st century, the use of herbal remedies for various diseases is a very common practice around the world [2]. In addition, many plant-based Chinese medicines have been found to be used both as medicines to treat illnesses and as food in culinary dishes, and star anise is one of them.

Star anise, a plant in the genus Anise in the Magnolia family, is mainly produced in China and Vietnam. The genus name *Illicium* is derived from the Latin word “illicere” meaning “allure”, indicating that the branches and leaves of star anise have an attractive fragrance [3]. The fruit of star anise is star shaped and it has an aromatic smell. It is an important medicinal plant and is also a commonly used as a spice. In China, the use of star anise as a spice can be traced back to the Song Dynasty (AD960–AD1279) and as a medicine to the Ming Dynasty (AD1368–AD1644). Qi in TCM refers to a very delicate substance in the human body, one of the basic components that make up the human body and maintain life activities. Yang (or Yang Qi) is a special and significant concept in TCM. It can warm the body and produce a feeling of excitement, thus allowing Qi and blood to flow smoothly. The Chinese Pharmacopoeia (2005 edition) documented the main effects star anise as warming Yang and dispersing cold, regulating Qi and relieving pain. As a well-known spice, star anise was first introduced to Europe in the seventeenth century, and it gets its distinctive licorice taste from the presence of star anise essential oil (SAO). It is one of the first traditional Chinese medicines announced by the Ministry of Health of the People’s Republic of China in 2002 for dual use in medicines and foodstuffs. In additions, it is also one of the Guangxi Zhuang Autonomous Region’s top ten characteristic Traditional Chinese Medicines, Daodi Chinese herbal materials and regional characteristic medicinal materials announced by the Guangxi Zhuang Autonomous Region Administration of Traditional Chinese Medicine (Gvangjsih Bouxcuengh Swcigih Cunghyihyoz Gvanjlijgiz) in 2020. Star anise has been used in China for more than a thousand years, and China is the only country in the world that can produce anise on a large scale.

Star anise is mainly found in East and Southeast Asia and southern North America. In East Asia, China is the main production area of star anise, followed by Vietnam, Cambodia, Myanmar, Sumatra in Indonesia, Kalimantan in the Philippines and other regions and countries. In North America, star anise is found mainly in Mexico, Haiti and Florida. In China, star anise oil, also known as “HuiYou” and “BaJiaoYou” is chemically diverse and has a wide range of uses. It is produced in China but is very popular in Malaysia, Vietnam, Indonesia and other regions (customs.gov.cn, accessed on 20 November 2022). Shikimic acid is the key synthetic precursor of Tamiflu (Roche Group, Basel, Switzerland) and is the only specific drug recommended by the International Health Organization to use against the H5N1 subtype. Star anise has a wide variety of pharmacological effects, including antioxidant, antibacterial, anti-inflammatory, insecticidal and antiviral properties. It is very similar to many plants, and confusing it with others has led to poisoning incidents.

The chemical composition of star anise is diverse, and the most important and widely studied components are SAO and shikimic acid. SAO is volatile oil that is extracted from star anise fruit. It has a unique anise flavor and is widely used in the food and pharmaceutical industries because of its antimicrobial and antioxidant properties. In addition to SAO, the other important compound in star anise, shikimic acid, is the main component of Tamiflu, which was developed by the Swiss Roche Group. Tamiflu is the only specific drug recommended by the International Health Organization to be used to treat the highly pathogenic avian influenza of the H5N1 subtype. Currently, the main industrial use of shikimic acid is for the synthesis of Tamiflu, and this has attracted widespread attention towards organic acids. Before 2000, shikimic acid was generally used as a chemical raw material to be converted into other chemicals and chemical reagents.

So far, a total of 201 chemical components have been isolated from star anise, including hydrocarbons, alcohols and esters, as well as natural products such as star anise oil, flavonoids, phenylpropanoids, organic acids phenols, terpenoids and others. These ingredients have been verified to have a variety of biological activities, and various extraction methods including steam distillation have been established for their extraction. This article reviews the general characteristics, chemical properties and biological activities of the constituents obtained from star anise, focusing particularly on SAO and shikimic acid. The outlook is that the research prospects of star anise are very broad and worthy of further research.

## 2. Botanical Description, Taxonomy and Geographic Distribution

### 2.1. Botanical Description and Taxonomy

*Illicium verum* Hook. F. is the English name of star anise, alias Illicium san-ki. It is a 10–15 m tall arbor plant. The leaves are either alternate or in 3–6 clusters of branches in a whirl at the top and are leathery or thick leathery, obovate-elliptical, oblique lance-shaped or oval, measuring approximately 50–150 mm long and 1–1.5 mm wide. The apex is short and acuminated or slightly obtuse-rounded, the upper midrib is slightly depressed or flat when fresh and the base is cuneate, with 4–6 pairs of lateral veins and 8–20 mm petioles. The flowers are pink to crimson, solitary in leaf axils or subterminal with a 15–40 mm long pedicel. The tepals number 7–12 and often exhibit inconspicuous translucent glandular dots. The largest tepal is broadly elliptical to broadly ovoid, 9–12 mm long and 8–12 mm wide. Its aggregated fruits tend to spread and are 35–40 mm in diameter, whereas the fruit stalks are 20–56 mm long. The seed pods number 7–8 and are 14–20 mm long with an apical rostrum and are obtusely rounded without apices. The seeds are brown and are 7–10 mm long. The star anise tree blooms twice a year, once in March–May with high yields and ripe fruits and again in August–October. According to the Flora of China, the type specimen of star anise was originally obtained from Kew Gardens, England, grown and propagated in Beihai, Guangxi Province, China [4,5]. The medicinal part of star anise is its fruit, which is picked in autumn and winter when the fruit turns from green to yellow [6]. Star anise is classified in the kingdom Plantae, phylum Angiospermae, class Magnoliopsida, order Austrobaileyales, family Magnoliaceae and genus *Illicium*. The fruit of star anise is cogwheel shaped and consists of an average of eight pods, and therefore it is generally called star anise. The “Huixiang” herbs include fennel, red fennel, star anise and cumin. As mentioned earlier, the genus name *Illicium* comes from the Latin word “illicere”, which can be translated as “to seduce and attract”, indicating that the fruits and branches of the plant have a seductive fragrance [3,4,5].

### 2.2. Geographic Distribution

Star anise is suitable for planting in deep, well-drained, fertile, moist and acidic sandy loam or loamy soil. It grows poorly in dry and barren or low-lying, waterlogged areas. It is mainly distributed in Southeast Asia and North America, of which Asia accounts for 80% of its source. In Southeast Asia, the main production area is China, followed by Vietnam, Cambodia and Myanmar (http://www.gxbajiao.org/, accessed on 13 June 2022). In China, star anise is mainly produced in Guangxi, including Baise, Nanning, Qinzhou, Wuzhou and Yulin, where it is mostly cultivated at an altitude of 200–700 m. In Yunnan province, including Funing, Guangnan, Xichou, Pingbian and Lvchun, it is mostly cultivated at its natural distribution levels, which are up to 1600 m in elevation [7]. The deputy director of the Anise Cinnamon Engineering Technology Research Center of the State Forestry Administration of China said that China’s production of star anise accounts for about 80% of the world’s production. Guangxi has the longest cultivation history and the most production, and in 2018, the Guangxi production of this plant accounted for more than 90% of the total national output. Guangxi has the reputation of being “the hometown of star anise” [8].

## 3. Pharmacology

### 3.1. Antimicrobial

The antimicrobial effect of star anise is one of the important focuses of modern pharmacological research. SAO has a wide inhibitory spectrum of activities against plant pathogenic fungi [5]. Additionally, some studies have shown that extracts from different parts such as the roots, branches, peels and leaves of star anise have certain antibacterial and antifungal activities. For four tested fungi (*Helminthosporiummaydis*, *Rhizoctonia cerealis*, *Helminthosporiurn carposaprum*, *Verticillium dahlia*), the antifungal rates of the seedpod and leaf extracts of *I. verum* were greater than 50%, and those for the root and branch extracts were lower than 50%. The antimicrobial activity of SAO (minimum inhibitory concentration, MIC = 0.5 µL/mL) for *Bacillus subtilis* was stronger than that of common preservatives, such as paraben [6].

For some fungi such as *Aspergillus flavus*, *Fusarium tricinctum* and *Candida albicans*, star anise also exhibits fungicidal characteristics, with MIC and MFC (minimum fungicidal concentration) values of 2.5–25 μL/mL [7]. Huang et al. determined the IC_50_ values for 11 plant pathogens (including *Alternaria solani, Bipolaris maydis* and *Botryodiplodia theobromae*) using a direct contact assay, and the IC_50_ values of SAO against mycelar growth ranged from 0.06 to 0.25 mg/mL. *Pythium aphanidermatum* and *Botryodiplodia theobromae* were selected to evaluate the antifungal activity of the vapor components the SAO from *I. verum* as well as that of *trans*-anethole by using the vapor contact assay. There was also a strong inhibition of *Magnaporthe oryzae* spore germination when using an inhibition assay, and the IC_50_ value of the oil was determined to be 0.32 mg/mL. At all concentrations in medium, *trans*-anethole displayed a very similar inhibitory rate to that of SAO against the test fungi, which suggested again that this was the main active component among the volatiles in the oil [8]. In accordance with these studies, Singh et al. used an inverted petri-plate technique, and they found that the volatile oil exhibited 100% zone inhibition for *Fusarium moniliforme*. It was also found to be highly effective in controlling the growth of *Penicillium citrium*, *Aspergillus flavus* and *Penicillium viridicatum* by exhibiting more than 75% mycelial zone inhibition, as well as 50% inhibition for *Aspergillus niger*.

In a 2020 report, Li et al. evaluated the apparent inhibitory effect of SAO on *A. flavus* by using a contact assay [9]. Mycelial growth was observed after 8 days of SAO treatments at 2.8 and 3.2 μL/mL. They tested the effects of SAO on *Aspergillus flavus* spore production using various concentrations and found that it was able to effectively inhibit spore production. Furthermore, the antifungal activities of SAO against *A. flavus* strains CGMCC 3.4408, CGMCC 3.4409 and CGMCC 3.4410 and their MIC and MFC values were determined. The results suggested that SAO exhibited a strong antifungal activity on the growth of the three *A. flavus* strains [10]. Recent findings from 2019 showed that SAO has antibacterial activity against *Penicillium*. Not only SAO but also some other anise extracts have similarly good antibacterial activities. A 2010 report stated that the supercritical CO_2_ and ethanol extracts of *I. verum* showed substantial antibacterial activities against 67 clinical drug-resistant isolates, including 27 *Acinetobacter baumannii*, 20 *Pseudomonas aeruginosa* and 20 methicillin-resistant *Staphylococcus aureus* strains [7,11,12].

Ibrahim et al. [12] used agar disc diffusion methods, agar plate dilution techniques and MIC and MBC (minimum bactericidal concentration) determinations to evaluate the antibacterial activity of star anise waste residue extract (SAWRE) against the most potent multi-drug-resistant strains of *Streptococcus pneumoniae*, *S. aureus, Klebsiella pneumoniae*, *A. baumannii*, *Escherichia coli* and *P. aeruginosa*. The results showed that SAWRE had significant antibacterial activities against all of the tested bacteria, with MIC values between 16 to 128 μL/mL. After binding to SAWRE, the MIC of cephradine, amoxicillin tetracycline and chloramphenicol was reduced by 512-, 64-, 8- and 2-fold, respectively, against *A. baumannii*. A combination of SAWRE and some antibiotics can represent a novel choice for the treatment of infectious diseases, as the waste extracts may act as activity-modifying agents for the antibiotics. The bacteriostatic mechanism of star anise is mainly through a variety of bacteriostatic components within the extracts. These act synergistically to degrade the bacterial cell walls and cause damage to the cytoplasmic membranes. They may also denature the membrane proteins, resulting in the loss of glucose, proteins and DNA from the cell, causing anabolic disorders and resulting in the death of bacteria and fungi [12].

The isolated compounds from *I. verum* were tested for anti-HIV activity by using an inhibition assay to assess their cytopathic effects on the virus. The compounds (-)-illicinone-A and 3,4-*seco*-(24*Z*)-cycloart-4(28),24-diene-3,26-doic acid, 26-methyl ester, which were isolated from star anise, were found to have anti-HIV activities, with EC_50_ values of 16.1 and 5.3 µM and with SI values of 18.2 and 15.6, respectively. In addition, it was also demonstrated that star anise extracts had antiviral activities against herpes simplex virus types 1 (HSV-1) and 2 (HSV-2) [13,14,15]. *I. verum* extracts exhibit excellent antiviral activity against infection with the viral strain SGIV-Gx. The antiviral effects of each type of *I. verum* extract were assessed using grouper spleen (GS) cells by Q2-AFMP and RT-qPCR. With both techniques, the results showed that the aqueous and ethanolic *I. verum* extracts displayed dose-dependent antiviral activities against grouper iridovirus. The activities of both extracts achieved >90% inhibition of viral growth. Determining the detailed antiviral mechanisms of *I. verum* extracts against SGIV-Gx infection is an important research direction for future studies [16]. In addition, (−)-bornyl *p*-coumarate isolated from star anise in 2022 was also found to have strong antiviral activity, with an IC_50_ of 1.74 μmol/L against influenza A H1N1 virus. This is better than that of Tamiflu (IC_50_ = 10.01 μmol/L) and ribavirin (IC_50_ = 10.76 μmol/L), and it might be considered a potential candidate in drug development for the treatment of influenza A virus (PR8) [16].

### 3.2. Antioxidant

The fruits of star anise are commonly used as spices, and these are an alternate source of antioxidants that has been used for a long time. The fine powder and extracts of star anise prepared using water and ethanol under normal or under supercritical CO_2_ conditions as well as SAO have all be found to have antioxidant properties. The antioxidant activity of star anise and its extracts were verified by adopting linoleic acid peroxidation, the *β*-carotene-linoleic acid system and DPPH (1,1-diphenyl-2-picryhydrazyl) radical-scavenging methods in different studies [17,18]. The addition of star anise fine powder to lemongrass oil enhances its oxidative stability, and together they form a natural antioxidant. The scavenging ability of the star anise antioxidant was evaluated by using the DPPH assay at various concentrations (500, 1000 and 1500 ppm) and was found to be 80.67, 81.38 and 81.73%, respectively [19]. The antioxidant activity of star anise against H_2_O_2_-induced DNA damage and human peripheral lymphocyte death was evaluated by assessing lipid peroxide inhibitory, hydroxyl-radical-scavenging, DPPH and superoxide free-radical-scavenging activities. The results showed that the water extracts of star anise had the most effective antioxidant activity. It was also shown that the aqueous extract of star anise acted as an antioxidant at a dose of 25 µg, and this amount provided protection to DNA against peroxides [20].

The DPPH-radical-scavenging method was also used by Yang et al. [21], who showed that the ethyl acetate extracts of star anise possess superior free-radical-scavenging abilities and reducing power compared to many other preparations. The strong correlation between its DPPH and TEAC values and those obtained from the reducing power assay implied that the antioxidants in the extracts were capable of scavenging free radicals and reducing different oxidants. Qualitative DPPH assays were performed on 25 essential oil products on TLC for 1–9 h at a temperature range of 30–70 °C. Two different areas of antioxidant activity with different polarities appeared on all the TLC plates. In addition, the highest antioxidant activity was observed for SAO when the sample was extracted at 60 °C for 1 day (EC_50_ value = 0.089 ± 0.05 mg/mL). According to numerous reports in the literature, the antioxidant activities of star anise and its extracts could be mainly due to the high polyphenol, carbohydrate, flavonoid and *trans*-anethole concentrations, along with a combined effect of all the phytochemicals [22]. At present, there is a search for new plant compounds that possess antioxidant activities, and this is a vital area of research in plant medicines. In general plant products are safe and are obtainable at relatively low cost [23,24].

### 3.3. Anti-Inflammatory

It is well documented that *I. verum* has anti-inflammatory properties. The anti-inflammatory effects of star anise aqueous extracts were investigated on xylene-induced auricle edemas in mice. The inhibition rate indicated that star anise significantly improved auricle edemas caused by xylene [24]. In another study, subjects in group A used a star aniseed-based mouthwash, and subjects in group B used a placebo (color-tinted water). This confirmed the effective anti-inflammatory properties of *I. verum* by recording gingival conditions before and after the intervention [25]. The anti-inflammatory activity of *I. verum* extract was also studied for its ability to inhibit protein denaturation, and the results were read spectrophotometrically. It was found to be effective at inhibiting heat-induced albumin denaturation at different concentrations of 100–500 µg/mL [26]. Five groups of six mice each were treated with distilled water (10 mL/kg), indomethacin (10 mg/kg) or methanolic, ethanolic or aqueous extracts (150, 250 and 350 mg/kg) of *I. verum*. Edema was induced in the lower metatarsals of the right hind paws of each animal, and the edema-associated paw thickness was measured after 1, 2, 3, 4 and 5 h by using a plethysmometer. The inflammation of paw volume in rats was significantly reduced in the methanol and ethanol *I. verum* extract test groups when compared to that in the control group. At present, star anise has a good therapeutic effect on acute inflammation such as that associated with the ear canal, oral cavity and airway surfaces, but whether it has an effect on other types of inflammation needs to be further researched [27].

### 3.4. Insecticidal and Anthelmintic

Phytochemical studies have shown that star anise tree wood is not subject to insect infestation. Using SAO as a grain storage protectant, it was found that the treatment of wheat or whole wheat flour with a 0.1% (*w*/*w*) dose of essential oil resulted in 100% inhibition of *Tribolium castaneum* (Herbest), *Rhizopertha dominica Fabricius* and *Tenebrio molitor Linne*. That meant a complete suppression of their reproduction. In addition, the egg dipping method also verified the toxic effects of SAO on the eggs of *Tenebrio molitor Linne* [28]. The results of the Kim et al. (2016) showed that the fumigation mortality of *Drosophila suzukii* by SAO could reach 80% within 24 h [29]. *Sogatella furcifera* was fed rice treated with *I. verum* extracted with n-hexane and methylene chloride. The results showed the treatment produced satisfactory insecticidal activity against *S. furcifera*, and this was concentration-dependent [30]. The repellent, antibacterial and insecticidal activities of anise were used to develop a coating material for food packaging, and the physical properties associated with the coating were improved by the addition of a reinforcing SAO filler in order to activate the coating solution. When packaging sliced wheat bread, the film exhibited a strong and long-lasting insect repellent activity, along with the ability to lower the microbial counts. In addition, it provided a better appearance when compared to the samples packaged with a control film [31]. The film prevented insects from approaching the bread as well as inhibiting the growth of microorganisms. Among four solvent-partitioned fractions [(1) n-hexane; (2) ether; (3) ethyl acetate; (4) water], the strongest repellency was found for the n-hexane fraction of star anise extract against *Plodia interpunctella* larvae [32]. This result is generally consistent with previous studies.

### 3.5. Other Activities

*I. verum* extracts affected the spontaneous activity as well as the sound and touch pain responses in mice at a dose of 200 mg/kg and produced moderate or slight depression [33,34]. This effect was produced by inhibiting the nociceptors, and this inhibition did not interfere with motor coordination [34]. Star anise processed products have the effects of warming Yang, dispersing cold and relieving pain [35]. *I. verum* extracts could increase exhaustive swimming and pole-climbing time periods as well as post exercise hepatic glycogen content in mice. They also raised lactate dehydrogenase activity and decreased lactate and serum urea nitrogen levels. These findings demonstrated that *I. verum* extracts have noticeable anti-fatigue effects on mice [36]. Good inhibition of aluminum corrosion in concentrated hydrochloric acid solution can be caused by *I. verum* extracts [37]. A mixture of chamomile and star anise has anti-motility effects and can decrease induced diarrhea in mice [38]. Furthermore, the results of a new study indicate that synthesized magnetite Fe_3_O_4_, spinel (2:1) and (4:1) NiFe_2_O_4_ using an extract of star anise as a green reducing agent showed high biomedical activities against liver carcinoma cells and non-small lung adenocarcinoma cells [39].

### 3.6. Toxicology

Star anise, one of the traditional Chinese herbs, has a long history. It was originally used as an ingredient in cooking, and later people gradually explored its medicinal value. According to previous literature, most cases of anise poisoning are due to accidental ingestion of anise analogs, except for cases of dietary overdose which were reported in the 19th century, but the exact mechanism of poisoning is not yet clear. In modern times, the first case of poisoning caused by over-consumption of anise was reported in 1992, in which the patient used star anise plants which were crushed to make cakes and suffered from paroxysmal vomiting, weakness and chills in the extremities after consumption [40]. In 1996, three new compounds extracted from the ethyl acetate extract of star anise, veranisatin A, B, C, were found to have convulsive effects and lethal toxicity at an oral dose of 3 mg/kg in mice, while anisatin caused similar effects at a dose of 1 mg/kg. In addition, several cases of poisoning have been reported in infants who took or were injected with anise, manifesting as neurological and gastrointestinal toxicity, but again the cause of the poisoning is not yet clear [41,42,43]. In 2004, it was speculated that, based on laboratory findings, the cause could be an excess of anise, contaminated anise, or a combination of both [44]. In 2016, a scholar using an aqueous extract of star anise seeds administered by gavage to mice showed that serial types of infusions caused transient behavioral changes consistent with neurological effects [45].

## 4. Phytochemistry

The chemical composition of star anise has been studied since 1983. Modern research has shown that different parts of this plant (including the roots, leaves and fruits) contain various chemical components, including a volatile oil, phenylpropanoids, sesquiterpene lactones and flavonoids. To date, 201 compounds have been identified, including organic acids, flavonoids, phenylpropanoids, lignans, sesquiterpenes, alcohols and some simple hydrocarbons. This includes 58 new compounds found more recently. Most of the new compounds found in the star anise are phenylpropanoid and lignan compounds. Chemical investigations of the genus *Illicium* have resulted in the isolation of prenylated C_6_–C_3_ compounds, neolignans and *seco*-prezizaane-type sesquiterpenes, which are characteristic chemical markers of this species. Phenylpropanoids and flavonoids are the most frequently reported components in the chemical composition studies of star anise and have a wide range of pharmacological effects. The chemical constituents that have been identified are listed in tables, and their corresponding structures are represented diagrammatically in the accompanying figures.

### 4.1. Star Anise Oil

The raw materials for the preparation of SAO are star anise fruits, branches and leaves, which are the main sources of aroma from these plants. The 2020 Chinese Pharmacopoeia classified SAO as a colorless or pale-yellow clear liquid, and its smell is similar to that of star anise. When cold, it often becomes turbid or will precipitate as crystals, and it turns clear after warming. It is readily soluble in 90% ethanol. This highly flavored volatile oil has long been the subject of research, as it is the main chemical component of star anise. SAO can also be used in food flavors such as alcoholic drinks, beverages, candy, baked goods and chewing gum and as a cigarette flavoring agent. It is also a good masking agent that can cover up unpleasant odors and is therefore, used in soap fragrances, mouth gargles and toothpaste. In the fragrance industry, one of its ingredients, anisole, is used to synthesize anisaldehyde, anisol, anisic acid and its esters. These monomer fragrances are widely used in toothpaste, foodstuffs, soap and cosmetics.

SAO has a long history, and it was originally produced as a commodity in Debao County, BaiSe City, Guangxi Province. Debao County was formerly known as “Tianbao”, so SAO has the alias of “Tianbao anise oil”. According to historical records, in the Ming Dynasty (1368–1644), the inhabitants of Debao began to plant anise trees. During the Xianfeng years (1851–1861), SAO, which has a high freezing point and excellent texture, was used as a pure fragrance. They began to steam and boil the leaves of star anise to make the oil. From 1912 to 1949, SAO was exported to Hong Kong, and it was later exported to more than 50 countries around the globe, including France, the United States, Japan and Canada. It is said that the world-famous “Paris perfume” has SAO as one of its ingredients, so there is a saying that “without SAO, the Paris perfume is not fragrant”.

Since 1995, China has maintained a foreign trade of star anise products. The main export areas are the United States, Africa and Europe. The average export volume of SAO is more than 600,000 kg each year. In 2003, the national output of SAO was 1,631,000 kg per year. China accounted for 80% of the total output, and the export volume accounted for 30% of the country’s total output. On 1 January 2010, the China and ASEAN Free Trade Area (CAFTA) was completed, forming a free trade area consisting of 11 countries and covering 1.9 billion consumers. Since then, China’s export of SAO has steadily increased, mainly to 10 countries and regions in Europe, Asia and North America.

The methods for extracting SAO from the fruits of star anise include traditional steam distillation (TSD), organic solvent extraction (OSE), headspace solid-phase microextraction (HSMS), microwave-assisted extraction (MAE), subcritical CO_2_ (SCOD) and supercritical CO_2_ fluid extraction (SCFE). The TSD method, which is easy to operate, low in cost and relatively stable in oil yield, has been a commonly used method for extracting SAO. SCFE is a new technology for chemical separation, and it has been widely used in recent years. Compared with the extraction of SAO by TSD, this technique operates at a lower temperature, which is close to ambient temperature but with a higher separation efficiency. In this method, the extraction and separation process are combined into one, and CO_2_ is used as the solvent, so no solvent residue is produced. Some experiments have shown that the chemical composition of TSD-obtained extracts is significantly reduced compared to that of SCFE and OSE. Extracts obtained using SCFE have a richer chemical composition, including various saturated and unsaturated fatty acids.

Due to the numerous and complex chemical constituents of SAO, most scholars have chosen GC-MS in order to explore its chemical constituents. The 158 chemical constituents identified in SAO are listed in Table 1. The most abundant component was found to be *trans*-anethole (>80%). SAO has been shown to possess broad-spectrum antibacterial activity. *Trans*-anethole has also been found in the essential oils of other plants, and it has been shown to possess insecticidal, larvicidal and antimicrobial activities. When the antifungal activity of *trans*-anethole was compared with that of SAO, the former was found to have similar inhibitory activity towards the test fungi, with IC_50_ values closed to those of the oil, with an average difference of 0.018. This suggested that *trans*-anethole is a major contributor to the antifungal properties of SAO [8].

### 4.2. Flavonoids

**Figure 1:**
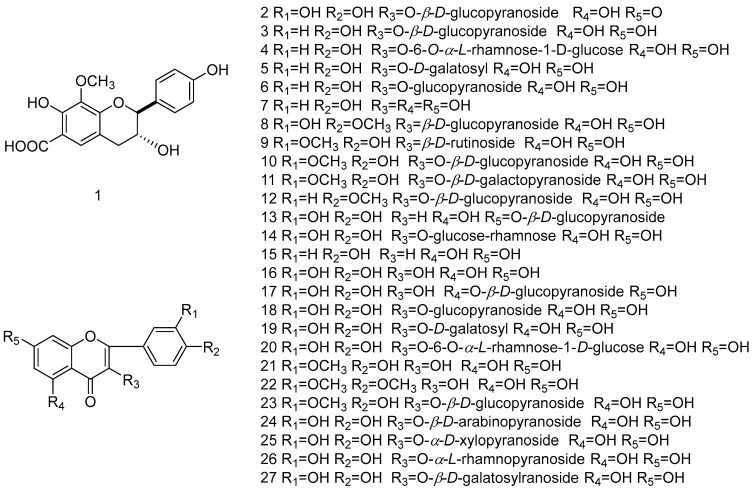
Chemical structures of compounds **1**–**27** from *Illicium verum*.

Flavonoids are widely distributed in plants, and most of them combine with sugars to form glycosides; this is also the case for those of *I. verum*. In addition to the common flavonoids—apigenin accidents (**15**) [56], star anise also contains high amounts of flavonoid glycosides [57,58,59] (**2**–**14**, **17**–**20** and **23**–**27**) and flavanols [60,61] (**16**, **21** and **22**). The basic parent nucleus of the flavonoids in star anise is a 2-phenyl-chromone. Quercetin, kaempferol and isorhamnetin are the common types of flavonoid aglycones in star anise, and together with D-glucose, D-xylose, L-rhamnose and other monosaccharides or rutinoses, they constitute the flavonoid glycosides found in the plants and its fruits. In 2015 [60], illiciumflavane acid (**1**), a new flavonoid acid, was discovered in the star anise fruits, and it was also found to have a significant inhibitory effect on A549 cancer cells with an IC_50_ value of 4.63 µM. The flavonoids isolated from *Illicium verum* are shown in Table 2, and chemical structure is shown in Figure 1.

### 4.3. Phenylpropanoids

Phenylpropanoids are a group of natural components containing one or more C_3_–C_6_ units, i.e., compounds consisting of benzene rings which are linked to three straight-chain carbons. The simple phenylpropanoids include phenylpropylene, phenylpropionic acid and phenylpropionaldehyde. There are a large number of C_3_–C_6_-type structural compounds in the genus Anise, and anisole (**33**) [63,64] was the first to be obtained from SAO produced from anise seeds in 1937. Later, many prenylated C_3_–C_6_ compounds were isolated from the fruits, leaves and roots of star anise. Most of the new compounds found in star anise are phenylpropanoids (**30**, **31**, **39**, **48**, **52**, **74**–**86**, **89**, **90**, **93**, **94**, **99**–**104**, **107**–**109, 115**–**120** and **124**–**131**) [61,65,66]. The phenylpropanoid compounds isolated from star anise have diverse structures. In addition to some simpler phenylpropanoids (**32**–**37**, **40**–**47**, **52**–**67**, **70** and **91**–**95**) [54,56,58,60,63,64,65,67,68,69,70], they also include some new phenylpropanoid glycosides formed by combining with glucopyranoside structures (**50**, **51**, **28**–**31**, **38**, **48**, **49**, **72**–**78**, **79**, **80** and **82**–**86**) [60,61,71]. Most of these phenylpropanoid compounds come from the fruits of star anise. (*E*)-1,4-bis(4-methoxyphenyl) but-3-en-2-one (**91**) [72] is obtained from the bottom of the distillation kettle of star anise and obtained by liquid separation and purification. 1-(4′-methoxyphenyl)-(1*R*,2*S* and 1*S*,2*R*)-propanediol (**50**) [61] exhibited the highest stability in a dose-dependent manner in an in vivo experimental model of tumor-necrosis-factor-induced septic shock, and this effect was accompanied by a reduction in plasma alanine aminotransferase levels.

**Figure 2:**
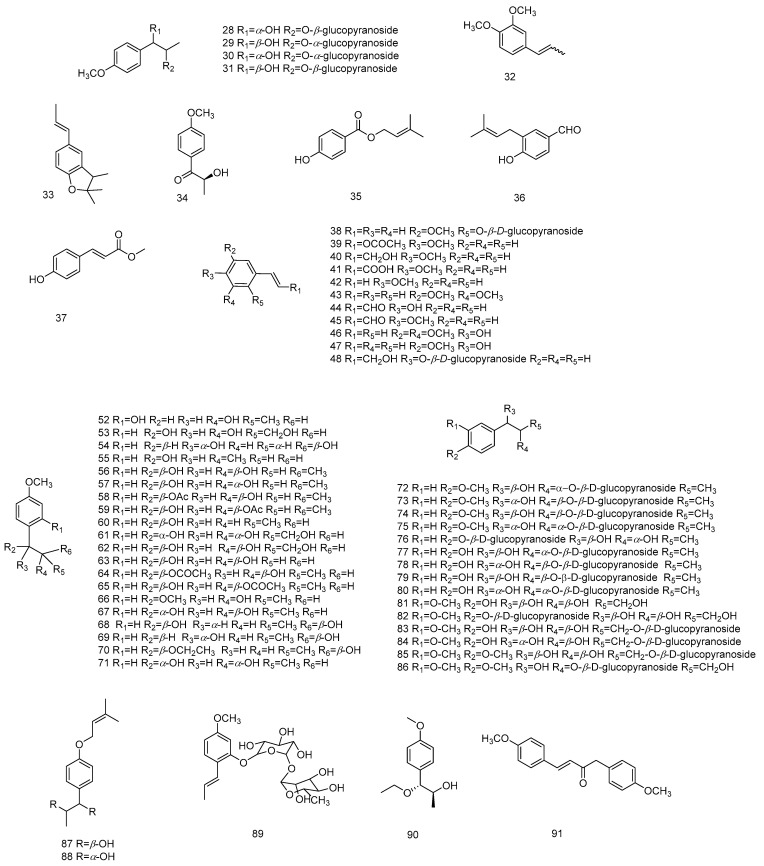
Chemical structures of compounds **28**–**91** from *Illicium verum*.

**Figure 3:**
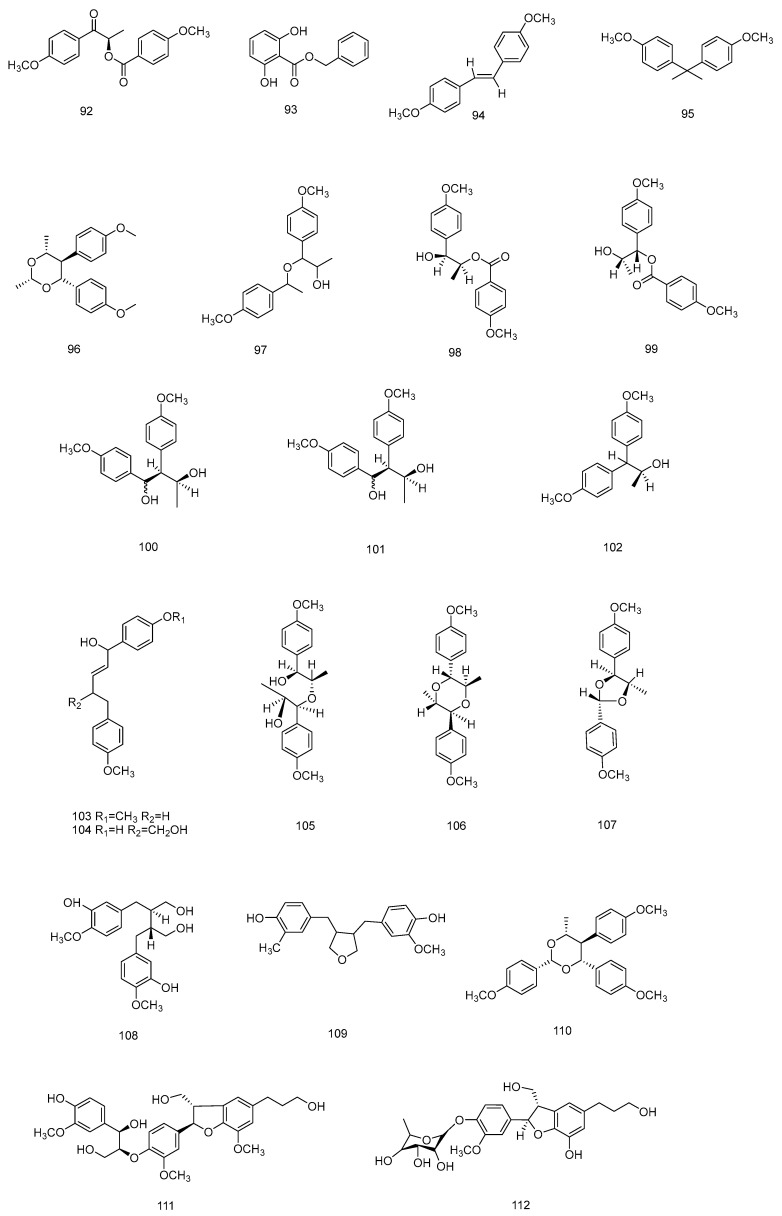
Chemical structures of compounds **92**–**112** from *Illicium verum*.

**Figure 4:**
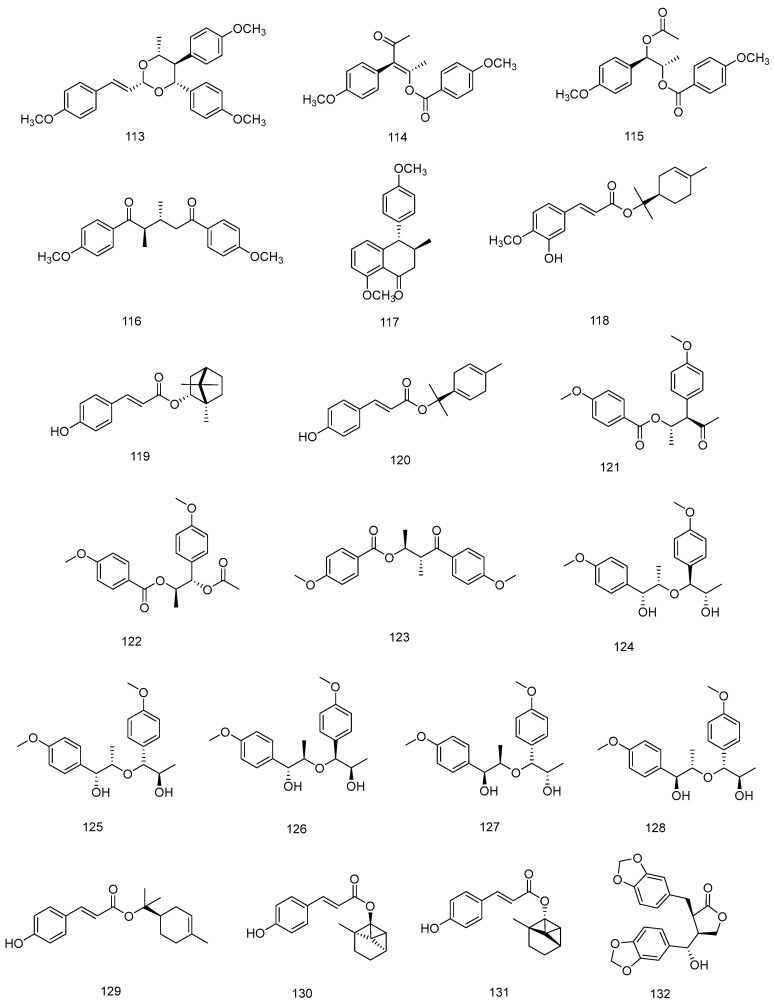
Chemical structures of compounds **113**–**132** from *Illicium verum*.

In 1998, Sy and Brown isolated novel lignans from anise leaves (**98**–**102** and **105**–**107**) [66], and later these compounds were also found in anise fruits [67,69]. The compounds verimol A (**98**) and harmandianone (**92**) showed concentration-dependent significant inhibition of lipopolysaccharide-induced tumour necrosis factor production in RAW264.7 cells [69]. Compounds **96**, **97**, **103**, **104**, **108**, **111** and **112** are known lignan-like compounds that are found in anise [67,73]. In 2022 [33], six new lignans and phenylpropanoid derivatives, illiciumiones AF (**114**–**118**) were isolated from the anise fruits. Although relatively few lignans have been found in anise so far, most of them are novel compounds. The phenylpropanoids isolated from *Illicium verum* are shown in Table 3, and chemical structure is shown in Figure 2, Figure 3 and Figure 4.

### 4.4. Organic Acids and Phenols

**Figure 5:**
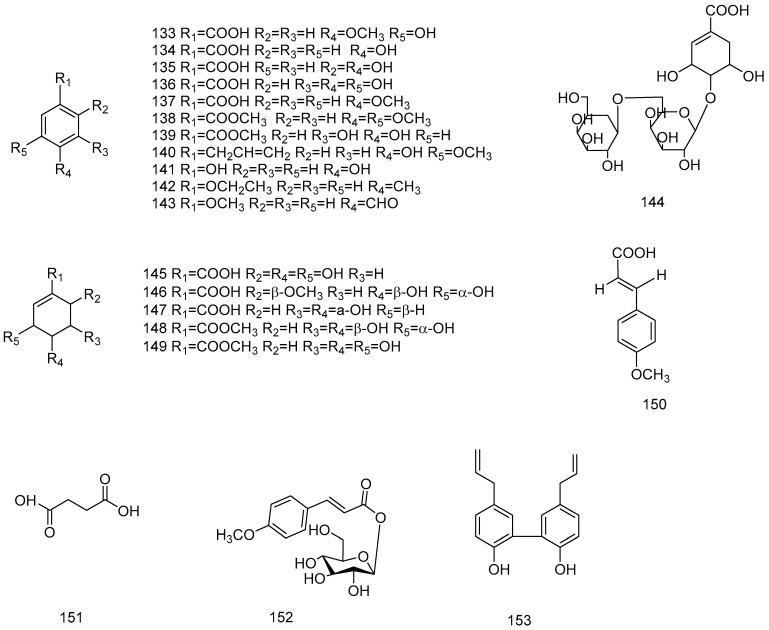
Chemical structures of compounds **133**–**153** from *Illicium verum*.

Organic acids and phenols in star anise include aliphatic organic acids (**144**–**149**, **151**) [58,60,69,73] and aromatic organic acids (**133**–**143**, **150**, **152**, **153**) [60,70,73]. All 20 organic acids are derived from the fruits of star anise. The composition of organic acids in star anise fruits is complex, and shikimic acid is the most important component. The organic acids and phenols isolated from *Illicium verum* are shown in Table 4, and chemical structure is shown in Figure 5.

#### Shikimic Acid

Shikimic acid is an aliphatic organic acid that was first isolated in 1885 from parts of the Japanese tree shikimino-ki (*I. anisatum*) [75,76] and later found to be present in the fruits of star anise. The extraction of shikimic acid from star anise was initiated following a global outbreak of avian influenza. Oseltamivir phosphate is the key component of Tamiflu and the only specific agent that has been recommended by the International Health Organization for the synthesis of antibodies against influenza A (H5N1). Shikimic acid is commonly used as the starting material for the industrial synthesis of oseltamivirphosphate [77]. In addition, shikimic acid has analgesic, antioxidant, anticoagulant, anti-inflammatory and antithrombotic activity as well as neuroprotective properties, and it is a precursor of antibacterial and anticancer agents [78].

**Figure 6:**
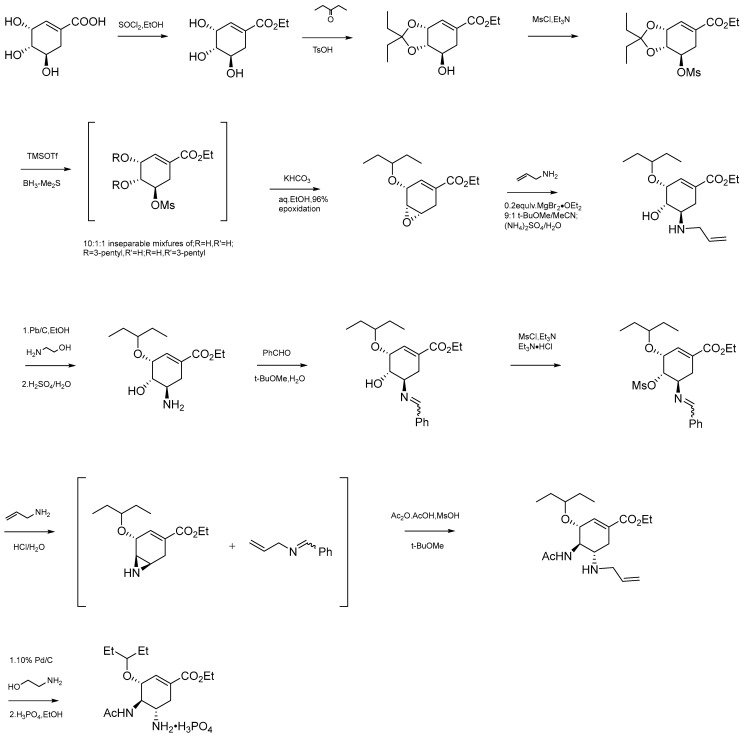
Roche’s published method for the synthesis of Tamiflu using shikimic acid [84].

Tamiflu played an important role in the fight against avian influenza attacks in 2005, and in October of that year, the World Health Organization and other international agencies recommended stockpiling the drug in preparation for a possible pandemic. At that time, the vast majority (90%) of the star anise produced in China was used by Roche to produce Tamiflu. Roche published the active ingredient of Tamiflu to be shikimic acid that was extracted from star anise and revealed a road map for the synthesis of Tamiflu, see Figure 6 below. So far, the presence of shikimic acid has been reported from *I. dunrlianum Tutch*, *I. henryi*, *I. temstroemioides*, *I. lanceolatum*, *I. simonsi* and other anise plants of the same genus [79,80,81,82]. In 2009, LC–UV and LC–MS methods were developed for the quantitative and qualitative analysis of shikimic acid. Nine different species of Illicium and 173 various plant extracts were tested for the presence of shikimic acid. The *Illicium Linn* plant had the highest content of shikimic acid, with some plants containing nearly 25 wt% shikimic acid in their dried fruits. Although shikimic acid exists in a large number of illicium plants and microorganisms, star anise is still the main raw material for its production worldwide due to its easy availability, abundant production and economic benefits [83]. Currently, the worldwide source of shikimic acid is star anise plants grown in China.

### 4.5. Terpenoids

A total of 23 terpenoids, including monoterpenes (**154**–**156**) [13,70,85], sesquiterpenes (**157**–**169**) [41,57,67,86], diterpenes (**170**–**173**) [67] and triterpenes (**174**–**176**), are currently [13,56,70], obtained from star anise. These include 2*α*,9*β*-dihydroxy-14 (10→1)-olivane-1(10)-ene) (**157**) [86] from the bottom of the anise distillation container, (E)-1-[(3-methylbut-2-enyl) oxy]-2-methoxy-4-(prop-1-enyl) benzene (**160**) [13] from the roots of anise and 11-O-denbenzoyl-11*α*-O-2-methylcyclopent-1-enecarboxyl tashironin A (**172**) [13], which are the new terpenoid compounds found. Among the family of Illiciaceae, the fruits of *Illicium anisatum* L. (shikimi in Japanese) are well known as neurotoxic and contain the convulsants anisatin and neoanisatin. However, these ingredients were not found in star anise until the 1990s. *Seco*-prezizaane-type sesquiterpenes have neurotoxicity [87] as well as anti-coxsackievirus B3 [88], anti-hepatitis B virus, neurotrophic [89] and other activities. These types of compounds are extremely rare in other plants but are characteristic of the star anise genus [90].

**Figure 7:**
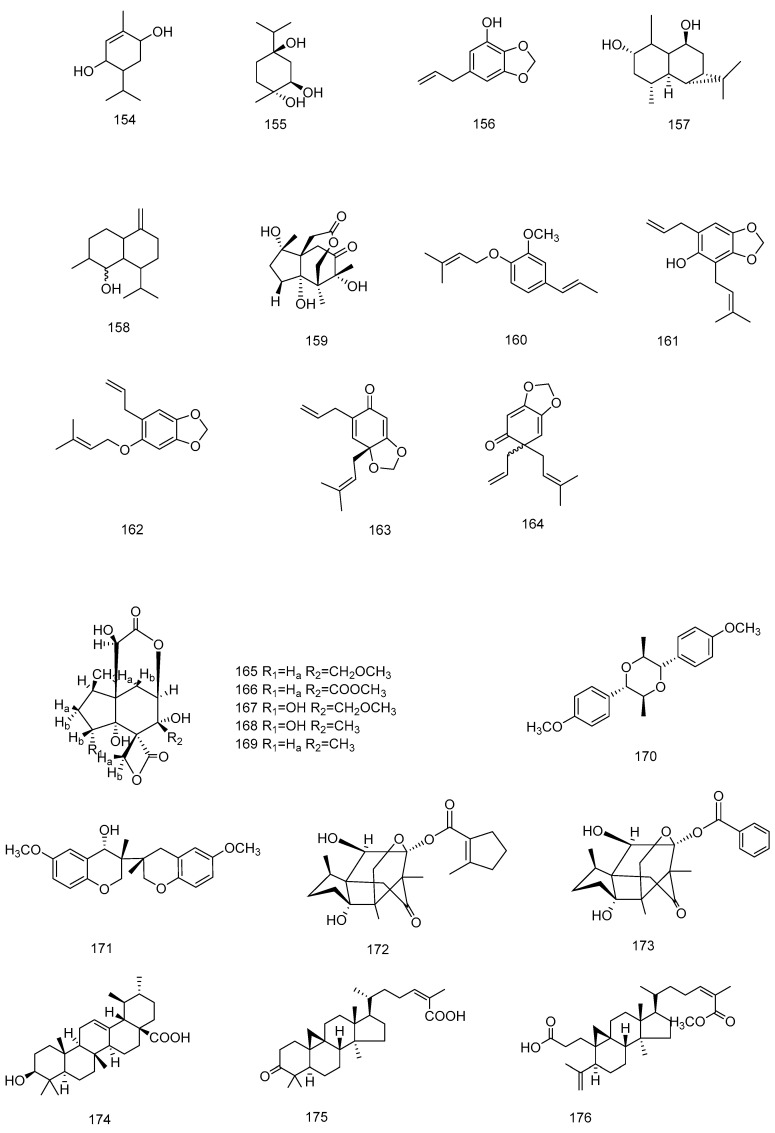
Chemical structures of compounds **154**–**176** from *Illicium verum*.

In 1996 [41], three new neurotoxic components, veranisatins A (**165**), B (**166**) and C (**167**), were isolated and identified from star anise, and preliminary pharmacological studies were carried out. The veranisatins led to convulsions and had lethal toxicities in mice at a dose of 3 mg/kg (p.o.), and at lower doses they caused hypothermia. Veranisatin A was tested for other pharmacological activities such as locomotor activity, as well as its effect as an analgesic. Veranisatin A decreased the locomotion enhanced by methamphetamine at oral doses of 0.1 mg/kg, and it demonstrated an analgesic effect on acetic acid-induced writhing and tail pressure pain in mice. 3,4-*seco*-(24*Z*)-cycloart-4(28),24-diene-3,26-dioic acid, 26-methyl ester (**176**) was found in both the roots and leaves of anise, and it as well as (-)-illicinole-A possessed moderate anti-HIV activities, with EC_50_ values of 16.0 and 5.1 μM and SI values of 18.2 and 15.6, respectively. When extracting and separating the chemical components of SAO after its hydrodistillation, the products were placed into a distillation kettle for single separations. Generally, the bottom liquid from the kettle was discarded as waste, but some groups analyzed the remaining residues in order to obtain a new olivetane-type sesquiterpene, 2*α*,9*β*-dihydroxy-14 (10→1) -olivane-1(10)-ene (1) (**157**), which was presumed to be from star anise. The terpenoids isolated from *Illicium verum* are shown in Table 5, and chemical structure is shown in Figure 7.

### 4.6. Other Constituents

**Figure 8:**
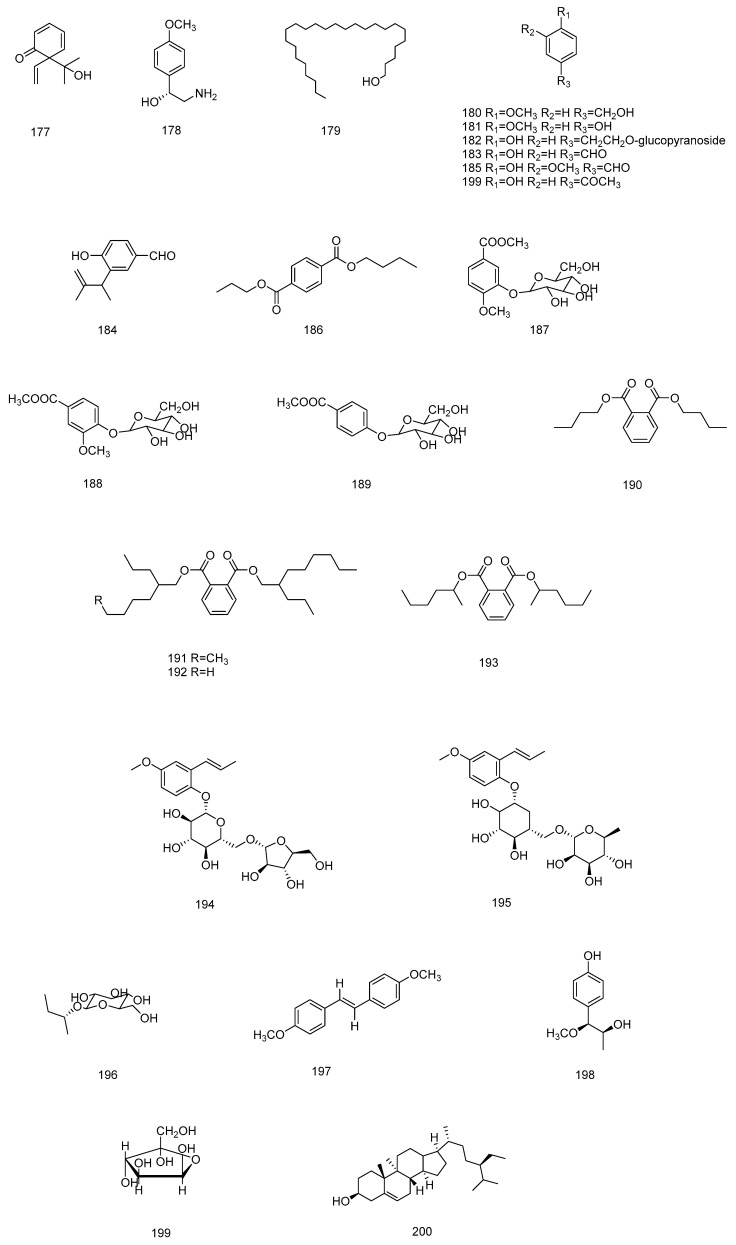
Chemical structures of compounds **177**–**200** from *Illicium verum*.

Some alcohols (**177**–**180**), phenols (**181**, **182**), aldehydes (**183**–**185**), esters (**186**–**193**), phenolic glycosides (**194**, **195**) and other compounds (**196**–**201**) with irregular chemical structures have also been isolated from star anise. The other constituents isolated from *Illicium verum* are shown in Table 6, and chemical structure is shown in Figure 8.

## 5. Modern and Traditional Uses of Star Anise

In the Song Dynasty (AD960–AD1279), star anise was widely produced in the region around Jiang in Guangxi, but at that time, people only knew that star anise was used as a condiment, not for medicinal purposes. Zhou Qufei wrote in his “*Ling Nan Dai Da*” (AD1178) that star anise should only be used as a soup blend and not as a medicine. By the Ming Dynasty (AD1368–AD1644), people not only knew that they could use star anise as a spice for seasoning but also that it could be used as a medicine. The use of star anise for a medicinal puposes was first published in the *Collected Essentials of Species of Materia*. (AD1505): “It is the size of a copper coin with 8 prongs and a seed inside each prong, and is used a lot in Chinese medicine”. *The Compendium of Materia Medica* (AD1578) recorded the morphological characteristics of star anise as split into eight petals, one petal and one nucleus, yellow-brown and sweet. This shows that star anise has been used in China for at least four hundred years. Modern monographs such as “Genuine and well-reputed medicinal materials in Guangxi”, “A list of plants in Nanning Arboretum, Guangxi” and “Modern Chinese Materia Medica” have all recorded that star anise has pharmacological effects such as elevating white blood cells while offering anti-bronchospasm and antibacterial properties. The compositions of 14 ancient classical formulas of star anise and 44 modern proprietary Chinese medicines using these plants are listed in Table 7 and Table 8.

Star anise is considered 95% edible and 5% medicinal. Star anise is widely used in some areas in Indonesia as a condiment for spicy cuisine. For instance, Aceh curry, Rendang Minang, Javanese cuisines and Balinese cuisines employ star anise. In northern China, star anise is one of the most popular seasonings, and it can be used directly in a variety of food preparations such as stewing, boiling, pickling, brining and soaking, or it can be processed into a five-spice seasoning powder [92]. Star anise extracts have the advantages of being natural, non-toxic and harmless, and they are usually used in the food industry to produce various food flavors. Star anise is also used in the daily chemical industry as a fragrant agent for perfumes, soaps, toothpastes and other cosmetics-associated products. It has certain developmental prospects as an essential oil fungicide, and it can be used as an antiseptic preservative for agricultural uses [93,94].

## 6. Quality Control

### 6.1. Studies on the quality standards of I. verum Hook. F.

Several new plants of the genus *Illicium Linn* have been discovered since 1982. In 2000, based on the study of more than 10,000 specimens of the genus *I. Linn* obtained from 120 herbarium collections in 18 countries and regions, Qi et al. proposed three new combinations. They confirmed that there were 34 species, 3 subspecies and 1 variety of plants of the *Illicium L.* worldwide. Among them, there were 9 species and 1 subspecies in the *Illicium* group and 25 species, 2 subspecies and 1 variety in the *I. verum* group. They also suggested that some species were morphologically difficult to distinguish [95,96]. *I. verum* is a medicinal as well as an edible plant and it has a wide distribution and grows in many places. It has a range of uses, and the quality standard of its medicinal composition has been constantly improved. The 2000 version of the Pharmacopoeia of the People’s Republic of China records the main identification methods of star anise. These are typically associated with a color reaction of its phloroglucinol to hydrochloric acid and its characteristics on TLC when compared with a standard star anise reference material. The volatility of its oil should not be less than 4.0% mL/g. In the 2005 version of the Pharmacopoeia of the People’s Republic of China, ultraviolet and visible spectrophotometry with a maximum absorption wavelength of 259 nm was added. The 2015 version of the Pharmacopoeia added that the *trans*-anisidine content should not be less than 4.0%. According to the latest 2020 edition of the Chinese Pharmacopoeia, the identification of star anise is mainly through trait identification, color reaction of resorcinol hydrochloric acid, and other methods that include a combination of the above methods [91].

In 2012 [97], Yuan et al. used TLC and HPLC analysis to establish quality control for star anise medicinal herbs. The extracted total and acid-insoluble ash products of star anise from 10 different origins were determined. At the same time, the active ingredients, shikimic acid and kaempferol, were identified in thin layers, and an HPLC quantitative study of the former was carried out. Their results provided a reference standard for the quality of star anise. The amounts of water- and alcohol-soluble extracts should not be less than 21.0 and 16.0%, respectively. In addition, the total and acid-insoluble ash content should not exceed 4.0 and 0.2%, respectively. The shikimic acid content should also not be less than 1.0%. This is used as a reference basis for formulating quantitative analysis standards for star anise medicinal materials. The methods used in this study were simple and accurate and were an effective control for the quality of star anise medicinal materials.

### 6.2. Extraction and Separation Methods

SAO is the main effective component of star anise, and anethole is the main volatile component of this oil. These two components are also the main standards for the quality control of *I. verum*. Therefore, optimizing their extraction is essential for ensuring the efficacy of this medical product. The extraction methods of SAO include TSD, Soxhlet extraction (SE), ultrasonic-assisted extraction (UE), SCFE, hydrodistillation (HD) and a combination microwave-assisted Soxhlet extraction method (MAEE). The extraction solvent generally includes absolute ethanol, petroleum ether and ethyl acetate. Different extraction solvents will not only change the composition of the extracts obtained but they can also alter the quality of the SAO as well as its physical properties. A ratio of solvent to sample of 17 mL/g, an extraction time of 16 min and a microwave power 505 Watts were found to be the optimal extraction conditions for using MAEE. The highest yield of SAO obtained was 24.98%, which was much higher than that from the SD method (7.17%) [35,98,99].

The conditions for TSD were optimized and it was concluded that the fruits must be fragmented to small size of less than 425 μm using a knife mill. For HD, the conditions reported as ideal were granulometry <425 μm, 8% mass, 1 h and a water volume of 200 mL. This process provided an SAO yield of 10.2% [99]. The conditions for UE were optimized as a solid-to-liquid ratio of 1:15 (g/mL), crushing particle size of 60 mesh and an ultrasonic time of 30 min [100]. Under the optional extract conditions, the extraction rate of SAO was determined to be 28.438 ± 0.11%. The optimum conditions for supercritical fluid CO_2_ extraction were extraction time of 1 h at a temperature of 40 °C and pressure of 250 bar when the yield of SAO was 14.5% [9]. The latest experiments compared SE, UE and DS methods, and the extraction rates of the different solvents used were ranked as ethanol > petroleum ether > ethyl acetate. Extracting the SAO using the different methods showed that the extraction yields were SE > UE > SD. The comparison of common extraction methods of star anise extract is shown in Table 9.

## 7. Conclusions

In this review, we have provided a critical analysis of the botanical properties, traditional uses, phytochemistry, pharmacology, quality control and toxicology of *Illicium verum*, which is used as a food and pharmaceutical product worldwide. Modern pharmacological studies have shown the plant components to have antioxidant, antibacterial, anti-inflammatory, insecticidal and antiviral effects. With an improvement in extraction techniques and the progressive advancement in pharmacological research, some success has been achieved in determining the chemical composition and pharmacological effects of this widely used plant.

## Figures and Tables

**Table 1:** Chemical components of star anise essential oil.

No.	Chemical Constituents	MF	References
1	2-acetonylcylohexanone	C_9_H_14_O_2_	[46]
2	limonene	C_10_H_l6_	[46]
3	*γ*-terpinen	C_10_H_l6_	[46]
4	*β*-linalool	C_10_H_18_O	[46]
5	spiro [4.5] dec-1-ene	C_10_H_l6_	[46]
6	1-terpinen-4-ol	C_10_H_18_O	[46]
7	3-undecyne	C_11_H_20_	[46]
8	*p*-allylanisole	C_10_H_12_O	[46]
9	*p*-cumic aldehyde	C_10_H_12_O	[46]
10	propanal, 2-methyl-3-phenyl	C_10_H_12_O	[46]
11	*trans*-anethole	C_10_H_12_O	[46]
12	benzaldehyde, 3-methoxy	C_8_H_8_O_2_	[46]
13	*p*-anisaldehyde	C_8_H_8_O_2_	[46]
14	anethole	C_10_H_12_O	[46]
15	anisold methyl ester	C_9_H_10_O_3_	[46]
16	anisyl aceton	C_10_H_12_O_2_	[46]
17	acetic acid, geraniol ester	C_12_H_20_O_2_	[46]
18	copaene	C_15_H_24_	[46]
19	iso-caryophyllene	C_15_H_24_	[46]
20	caryophyllene	C_15_H_24_	[46]
21	2-norpinene,2,6-d	C_15_H_24_	[46]
22	*β*-famesene	C_15_H_24_	[46]
23	benzene, 1,2-dimethoxy-4-(1-propenyl)	C_11_H_14_O_2_	[46]
24	benzenemethanol, 2-(2-aminopropoxy)-3-methyl	C_11_H_17_NO_2_	[46]
25	bicyclo [3,1,1] hept-2-ene, 2-ethanol, 6,6-dimethyl	C_11_H_18_O_2_	[46]
26	bicyclo [3,1,1] hept-2-ene, 2,6-dimethyl-6-(4-methyl-3-pentenyl)	C_15_H_24_	[46]
27	*γ*-elermene	C_15_H_24_	[46]
28	*α*-famesene	C_15_H_24_	[46]
29	cyclohexene, 1-methyl-4-(5-methyl-l-methylene-4-hexenyl)	C_15_H_24_	[46]
30	germacrene D	C_15_H_24_	[46]
31	phenylethanolamine	C_8_H_11_NO	[46]
32	acethydrazide	C_2_H_6_N_2_O	[46]
33	surfynol 102	C_12_H_22_O_2_	[46]
34	*p*-anisoin	C_16_H_16_O_4_	[46]
35	*trans*-nerolidol	C_15_H_26_O	[46]
36	1-(3-methyl-2-butenoxy)-4-(1-propenyl) benzene	C_14_H_18_O	[46]
37	bicyclo [2,2,1] heptane-2,3-dione, 6-(acetyloxy)-1,5,5-trimethyl, endo	C_12_H_16_O_4_	[46]
38	hydrazinecarboxylic acid, ethyl ester	C_3_H_8_N_2_O_2_	[46]
39	propanoic acid, 2-methyl, 3, 7-dimethyl-2, 6-octadienyl ester(*E*)	C_14_H_24_O_2_	[46]
40	*p*-allylphen	C_9_H_10_O	[46]
41	hexyl oleate	C_24_H_46_O_2_	[46]
42	*α*-pinene	C_10_H_16_	[47]
43	cyclotetrasiloxane, octamethyl-	C_8_H_24_O_4_Si_4_	[47]
44	myrcene	C_10_H_16_	[47]
45	*α*-phellandrene	C_10_H_16_	[47]
46	*δ*-3-carene	C_10_H_16_	[47]
47	sabinene	C_10_ H_16_	[47]
48	1,8-cineole	C_10_H_18_O	[47]
49	linalool	C_10_H_18_O	[47]
50	*L*-menthone	C_10_H_18_O	[47]
51	4-terpineol	C_10_H_18_O	[47]
52	estragole	C_10_H_12_O	[47]
53	benzene, 1-methoxy-4-(1-propenyl)-	C_10_H_12_O	[47]
54	*trans*-ciminnamaldehyde	C_9_H_8_O	[47]
55	*β*-caryophyllene	C_15_H_24_	[47]
56	*trans-α*-bergamotene	C_15_H_24_	[47]
57	iso-caryophillene	C_15_H_24_	[48]
58	*β*-pinene	C_10_H_l6_	[48]
59	*β*-myrcene	C_10_H_l6_	[48]
60	Δ^3^-carene	C_10_H_l6_	[48]
61	iso-terpinene	C_10_H_l6_	[48]
62	*p*-cymene	C_10_H_l4_	[48]
63	ocimene	C_10_H_l6_	[48]
64	*α*-terpineol	C_10_H_12_O	[48]
65	cis-anethole	C_10_H_12_O	[48]
66	anisaldehyde	C_8_H_8_O_2_	[48]
67	*α*-copaene	C_15_H_24_	[48]
68	anisketone	C_10_H_12_O_2_	[48]
69	elemene	C_15_H_24_	[48]
70	*α*-bergamotene	C_15_H_24_	[48]
71	(*Z, E*)-*α*-famesene	C_15_H_24_	[48]
72	(*E, E*)-*α*-famesene	C_15_H_24_	[48]
73	humulene	C_15_H_24_	[48]
74	methyl isoeugenol	C_11_H_14_O_2_	[48]
75	geranyl valerate	C_15_H_26_O_2_	[48]
76	*β*-bisabolene	C_15_H_24_	[48]
77	*β*-cadinene	C_15_H_24_	[48]
78	feniculine	C_14_H_18_O	[48]
79	cayopyllene oxide	C_15_H_26_O	[48]
80	*α*-cadinol	C_15_H_26_O	[48]
81	2,6,10-trimethyltetradecane	C_17_H_36_	[48]
82	1-methoxy-4-(1-methyl-2-propenyl) benzene	C_11_H_14_O	[48]
83	1-(3-methyl-2-butenoxy)-4-(1-propenyl) benzene	C_14_H_18_O	[48]
84	hexadecanoic acid	C_16_H_32_O_2_	[48]
85	octadeca-9C,12C,15C trienoic acid	C_18_H_30_O_2_	[48]
86	octadeca-9C,12C dienoic acid	C_18_H_30_O_2_	[48]
87	octadeca-9C-enoic acid	C_18_H_34_O_2_	[48]
88	octadecanoic acid	C_18_H_36_O	[48]
89	camphene	C_10_H_16_	[49]
90	*α*-fenchene	C_10_H_16_	[49]
91	eucalyptol	C_10_H_8_O	[49]
92	1*R*-*α*-pinene	C_10_H_16_	[49]
93	terpinolene	C_10_H_16_	[49]
94	cis-linalool oxide	C_10_H_18_O_2_	[49]
95	2,4-dimethylanisole	C_9_H_12_O	[49]
96	fenchyl acetate	C_12_H_20_O_2_	[49]
97	2,5-dimethyl-3-viny-1,4-hexadiene	C_10_H_6_	[49]
98	*α*-bisabolene	C_15_H_24_	[49]
99	*α*-cedrene	C_15_H_24_	[49]
100	terpinen-4-ol	C_10_H_18_	[49]
101	dihydro anethol	C_10_H_14_O	[49]
102	iso-boenyl fomate	C_11_H_8_O_2_	[49]
103	*γ*-muurolene	C_15_H_24_	[49]
104	*α*-himachalene	C_15_H_24_	[49]
105	geimacrene D	C_15_H_24_	[49]
106	2,3,4,5,6-pentmethylbenzaldehyde	C_12_H_16_O	[49]
107	benzalacetone	C_10_H_10_O	[49]
108	cubenol	C_15_H_26_O	[49]
109	1,2,8,9-diepoxy-p-menthane	C_10_H_16_O_2_	[49]
110	caryophyllene oxide	C_15_H_24_O	[49]
111	cis-*α*-santalol	C_15_H_24_O	[49]
112	anisic aldehyde	C_8_H_8_O_2_	[49]
113	germacrene D-4-ol	C_15_H_26_O	[49]
114	*α*-guaiene	C_15_H_24_	[49]
115	methyl p-anisate	C_9_H_10_O_3_	[49]
116	iso-anethole	C_10_H_12_O	[49]
117	Z-*α*-*trans*-bergamotol	C_15_H_24_O	[49]
118	spathulenol	C_15_H_24_O	[49]
119	acetylanisole	C_9_H_10_O_2_	[49]
120	cinnamyl acetate	C_11_H_12_O_2_	[49]
121	*p*-acetonyl anisole	C_10_H_12_O_2_	[49]
122	tau-cadinol	C_15_H_26_O_2_	[49]
123	*p*-methoxypropiophencne	C_10_H_12_O_2_	[49]
124	muurolol	C_15_H_26_O	[49]
125	2-methoxy-*α*-methyl-benzeneenthanol	C_10_H_14_O_2_	[49]
126	foeniculine	C_14_H_8_O	[49]
127	cinnamylalcohol	C_9_H_10_O	[49]
128	4-methoxy-benzenepropanol	C_10_H_14_O_2_	[49]
129	7-isopropyl-1,4,4a-trimethyl-1,2,3,4,4a,9,10,10a-octaphydrophenanthrene	C_20_H_30_	[49]
130	acetocumene	C_12_H_8_	[49]
131	3-methoxycinnamaldehyde	C_10_H_10_O_2_	[49]
132	methylp-methoxycinnamate	C_11_H_13_O_3_	[49]
133	ethylp-methoxycinnamate	C_12_H_5_O_3_	[49]
134	1,5,5-trimethyl-6-acetymethyl-cyclohexene	C_12_H_20_O	[49]
135	anisic alcohol	C_8_H_10_O_2_	[49]
136	iso-eugenol	C_10_H_12_O_2_	[49]
137	oleic acid	C_18_H_34_O_2_	[49]
138	*β*-phellandrene	C_10_H_16_	[50]
139	1,4-cineole	C_10_H_18_O	[51]
140	terpinen-1-ol	C_10_H_18_O	[10]
141	*γ*-terpinene	C_10_H_16_	[50]
142	*α*-terpinene	C_10_H_16_	[10]
143	*γ*-terpineol	C_10_H_18_O	[50]
144	geraniol	C_10_H_18_O	[52]
145	cis-*β*-ocimene	C_10_H_16_	[50]
146	*trans-β*-ocimene	C_10_H_16_	[50]
147	borneol	C_10_H_18_O	[50]
148	*δ*-elemene	C_15_H_24_	[50]
149	*β*-elemene	C_15_H_24_	[50]
150	geranyl acetate	C_12_H_20_O_2_	[50]
151	*p*-anisic acid methyl ester	C_9_H_10_O_3_	[50]
152	*δ*-cadinene	C_15_H_24_	[50]
153	*α*-cubebene	C_15_H_24_	[53]
154	anisylacetone	C_11_H_14_O_2_	[7]
155	*α*-muurolene	C_15_H_24_	[7]
156	carryophyllene oxide	C_15_H_24_O	[53]
157	*trans*-chalcone	C_15_H_12_O	[54,55]
158	4-methoxy-benzaldehyde	C_8_H_8_O_2_	[54,55]

**Table 2:** The flavonoids isolated from *Illicium verum*.

No.	Chemical Constituents	Parts	References
1	illiciumflavane acid	Fruit	[60,62]
2	isoquercitrin	Fruit	[57]
3	kaempferol-3*-O-β-D*-glucopyranoside	Fruit	[58]
4	kaempferol-3-*O*-rutinoside	Fruit	[58]
5	kaempferol-3-*O*-galactosyl	Fruit	[58]
6	kaempferol-3-*O*-glucopyranoside	Fruit	[58]
7	kaempferol	Fruit	[58]
8	tamarixetin 3-*O*-neohesperidoside	Fruit	[57]
9	isorhamnetin-3-*O*-ruti noside	Fruit	[57]
10	isorhamnetin-3*-O-β-D*-glucopyranoside	Fruit	[59]
11	isorhamnetin-3*-O-β-D*-galactopyranoside	Fruit	[59]
12	acacetin-3*-O-β-D*-glucopyranoside	Fruit	[58]
13	luteolin-7*-O-β-D*-glucopyranoside	Fruit	[59]
14	rutin	Fruit	[59]
15	apigenin	Fruit	[56]
16	quercetin	Fruit	[52]
17	quercetin-5*-O-β-D*-glucopyranoside	Fruit	[58]
18	quercetin-3-*O*-glucopyranoside	Fruit	[58]
19	quercetin-3-*O*-galactosyl	Fruit	[58]
20	quercetin-3-*O*-rutinoside	Fruit	[58]
21	3′-methoxy quercetin	Fruit	[60]
22	3′,4′-dimethoxy quercetin	Fruit	[60]
23	quercetin 3′-*O*-methyl-3*-O-β-D*-glucopyranoside	Fruit	[57]
24	quercetin-3-*O-α-L*-arabinopyranoside	Fruit	[57]
25	quercetin-3-*O-D*-xylopyranoside	Fruit	[57]
26	quercetin-3-*O-α-L*-rhamnopyranoside	Fruit	[59]
27	quercetin-3*-O-β-D*-galactopyranoside	Fruit	[59]

**Table 3:** The phenylpropanoids isolated from *Illicium verum*.

No.	Chemical Constituents	Parts	References
28	1-(4′-methoxyphenyl)-(1*S*,2*R*)-propan-1-ol 2*-O-β-D*-glucopyranoside	Fruit	[61]
29	1-(4′-methoxyphenyl)-(1*R*,2*S*)-propan-1-ol 2*-O-β-D*-glucopyranoside	Fruit	[61]
30	1-(4′-methoxyphenyl)-(1*S*,2*S*)-propan-1-ol 2*-O-β-D*-glucopyranoside	Fruit	[61]
31	1-(4′-methoxyphenyl)-(1*R*,2*R)*-propan-1-ol 2*-O-β-D*-glucopyranoside	Fruit	[61]
32	(*E*)-1,2-dimethoxy-4-propenyl benzene	Root	[56]
33	anisoxide	Root	[63,64]
34	(2*R*)-2-hydroxy-1-(4-methoxyphenyl)-1-propane	Fruit	[67]
35	3-methylbut-2-enyl-4-hydroxybenzoate	Fruit	[33]
36	4-hydroxy-3-(3-methyl-2-buten-1-yl) benzaldehyde	Fruit	[33]
37	(*E*)-methyl *p*-coumarate	Fruit	[33]
38	4-methoxy-2-(*E*)-propenylphenyl-*β-D*-glucopyranoside	Fruit	[60]
39	verimol I	Fruit	[66]
40	3-(*p*-methoxyphenyl)-2-propen-1-ol	Fruit	[66]
41	3-(4-methoxyphenyl)-2-(*E*)-propenoic-acid	Fruit	[67]
42	1-methoxyphenyl)-4-propenylbenzene	Fruit	[67]
43	*trans*-(3,5-dimethoxyphenyl)-propene	Fruit	[67]
44	*trans-p*-coumary aldehyde	Fruit	[69]
45	*E*-4-methoxycinnamaldehyde	Fruit	[69]
46	methoxyeugenol	Root	[56]
47	eugenol	Root	[56]
48	(*Z*)-4-hydroxycinnamyl alcohol 4*-O-β-D*-glucopyranoside	Fruit	[71]
49	(*E*)-4-hydroxycinnamyl alcohol 4*-O-β-D*-glucopyranoside	Fruit	[71]
50	1-(4′-methoxyphenyl)-(1*R*,2*S* and 1*S*,2*R*)-propanediol	Fruit	[61]
51	1-(4′-methoxyphenyl)-(1*R*,2*R* and 1*S*,2*S*)-propanediol	Fruit	[61]
52	verimol J	Fruit	[66]
53	1-(4′-methoxyphenyl)-1,2,3-trihydroxypropane	Fruit	[65]
54	(1*R*,2*S*)-1-(4′-methoxyphenyl)-propanediol	Fruit	[60]
55	1′-(1-methoxyphenyl)-1′-propanol	Fruit	[70]
56	(1*R*,2*R*)-1-(4-methoxyphenyl) propane-1,2-diol	Fruit	[67]
57	(1*S*,2*R*)-1-(4-methoxyphenyl) propane-1,2-diol	Fruit	[67]
58	(±) -(1*R*,2*R*)-2-hydroxy-1-(4-methoxyphenyl) propyl acetate	Fruit	[67]
59	(±) -(1*R*,2*R*)-1-hydroxy-1-(4-methoxyphenyl) propyan-2-yl-acetate	Fruit	[67]
60	1-(4-methoxyphenyl)-1-propanol	Fruit	[67]
61	(1*S*,2*S*)-1-(4-methoxyphenyl)-1,2,3-propanetriol	Fruit	[67]
62	(1*R*,2*R*)-1-(4-methoxyphenyl)-1,2,3-propanetriol	Fruit	[67]
63	4-(4-methoxyphenyl)-2,2,5-trimethyl-1,3-dioxolane	Fruit	[67]
64	2-acetoxy-1-(*p*-methoxyphenyl) propsn-1-ol	Fruit	[69]
65	1-acetoxy-1-(*p*-methoxyphenyl) propan-2-ol	Fruit	[69]
66	1-methoxy-1-(4-methoxyphenyl)-2-propanol	Fruit	[69]
67	1-(4′-methoxyphenyl)-(1*R*,2*S*)-propane diol	Fruit	[69]
68	*thero*-anethole glycol	Fruit	[67]
69	*orythro*-anetholeglycol	Fruit	[67]
70	(1*S*,2*R*)-1-ethoxy-1-(4-methoxyphenyl) propan-2-ol	Fruit	[33]
71	(1*R*,2*R*)-1-(4-methoxyphenyl) propane-1,2-diol	Fruit	[74]
72	(1′*R*,2′*S*)-anethole glycol 2′*-O-β-D*-glucopyranoside	Fruit	[71]
73	(1′*S*,2′*R*)-anethole glycol 2′*-O-β-D*-glucopyranoside	Fruit	[71]
74	(1′*R*,2′*R*)-anethole glycol 2′*-O-β-D*-glucopyranoside	Fruit	[71]
75	(1′*S*,2′*S*)-anethole glycol 2′*-O-β-D*-glucopyranoside,	Fruit	[71]
76	an equivalent mixture of two stereoisomeric erythro-1′-(4-hy- droxyphenyl) propane-1′,2′-diol 4*-O-β-D*-glucopyranosides	Fruit	[71]
77	(1′*R*,2′*S*)-1′-(4-hydroxyphenyl) propane-1′,2′-diol 2′*-O-β-D*-glucopyranoside	Fruit	[71]
78	(1′*S*,2′*R*)-1′-(4-hydroxyphenyl) propane-1′,2′-diol 2′*-O-β-D*-glucopyranoside	Fruit	[71]
79	(1′*R*,2′*R*)-1′-(4-hydroxyphenyl) propane-1′,2′-diol 2′*-O-β-D*-glucopyranoside	Fruit	[71]
80	(1′*S*,2′*S*)-1′-(4-hydroxyphenyl) propane-1′,2′-diol 2′*-O-β-D*-glucopyranoside	Fruit	[71]
81	(1′*R*,2′*R*)-guaiacyl glycerol	Fruit	[71]
82	(1′*R*,2′*R*)-guaiacyl glycerol 4*-O-β-D*-glucopyranoside	Fruit	[71]
83	(1′*R*,2′*R*)-guaiacyl glycerol 3′*-O-β-D*-glucopyranoside	Fruit	[71]
84	(1′*S*,2′*R*)-guaiacyl glycerol 3′*-O-β-D*-glucopyranoside	Fruit	[71]
85	(1′*R*,2′*R*)-4-O-methylguaiacyl glycerol 3′*-O-β-D*-glucopyranoside	Fruit	[71]
86	4-*O*-methylguaiacyl glycerol 2′*-O-β-D*-glucopyranoside	Fruit	[71]
87	(7*S*,8*S*)-7-(4-((3-methylbut-2-en-1-yl) oxy) phenyl) propane-7,8-diol	Fruit	[74]
88	(7*R*,8*R*)-7-(4-((3-methylbut-2-en-1-yl) oxy) phenyl) propane-7,8-diol	Fruit	[74]
89	(*E*)-2-(prop-1-enyl)-5-methoxyphenol-1-*O-α-L*-rhamnopyranosyl-(1→6)*-O-β-D*-glucopyranoside	Fruit	[65,66]
90	(1*S*,2*R*)-1-ethoxy-1-(4-methoxyphenyl) propan-2-ol	Fruit	[74]
91	(*E*)-1,4-bis (4-methoxyphenyl) but-3-en-2-one	Fruit	[72]
92	harmandianone	Fruit	[69]
93	verimol K	Fruit	[66]
94	(*E*)-1,2-bis(4-methoxyphenyl) ethene	Fruit	[69]
95	1-biphenyl-2,2′-dimethoxy-5,5′-di-2-propenyl	Fruit	[67]
96	anemonenorin B	Fruit	[33]
97	verimol O	Fruit	[67]
98	verimol A	Leaf	[66]
99	verimol B	Leaf	[66]
100	verimol D	Leaf	[66]
101	verimol E	Leaf	[66]
102	verimol F	Leaf	[66]
103	verimol L	Fruit	[67]
104	verimol M	Fruit	[67]
105	verimol G	Leaf	[66]
106	verimol H	Leaf	[66]
107	verimol C	Leaf	[66]
108	secoisolariciresinol	Fruit	[67]
109	4-((4-(4-hydroxy-3-methoxybenzyl) tetrahydrofuran-3-yl) methyl)-2-methylphenol	Leaf	[66]
110	anemonenorin A	Fruit	[33]
111	xanthiumnolic C	Fruit	[73]
112	icariside E4	Fruit	[73]
113	illiciumiones A	Fruit	[33]
114	illiciumiones B	Fruit	[33]
115	illiciumiones C	Fruit	[33]
116	illiciumiones D	Fruit	[33]
117	illiciumiones E	Fruit	[33]
118	illiciumiones F	Fruit	[33]
119	(−)-bornyl *p*-coumarate	Fruit	[33]
120	(*R*)-2-(4-methylcyclohex-3-en-1-yl) propan-2-yl (*E*)-3-(4-hydroxyphenyl) acrylate	Fruit	[33]
121	(2*α*,3*β*)-3-(4-methoxyphenyl)-4-oxopentan-2-yl 4-methoxybenzoate	Fruit	[74]
122	(7*α*,8*α*)-7-acetoxy-7-(4-methoxyphenyl) propan-8-yl-4-methoxybenzoate	Fruit	[74]
123	(8*α*,9*β*)-1-(4-methoxyphenyl)-8,9-methyl-9-methoxybenzoate	Fruit	[74]
124	verimol O	Fruit	[74]
125	verimol P	Fruit	[74]
126	verimol Q	Fruit	[74]
127	verimol R	Fruit	[74]
128	verimol S	Fruit	[74]
129	(*R*)-2-(4-methylcyclohex-3-en-1-yl) propan-2-yl (*E*)-3-(4-hydroxyphenyl) acrylate	Fruit	[74]
130	(-)-bornyl *p*-coumarate	Fruit	[74]
131	(+)-bornyl *p*-coumarate	Fruit	[74]
132	(7′*S*)-parabenzlactone	Fruit	[74]

**Table 4:** The organic acids and phenols isolated from *Illicium verum*.

No.	Chemical Constituents	Parts	References
133	3-hydroxy-4-methoxy benzoic acid	Fruit	[60]
134	*p*-hydroxy benzoic acid	Fruit	[60]
135	2,4-dihydroxy benzoic acid	Fruit	[60]
136	gallic acid	Fruit	[60]
137	*p*-methoxy benzoic acid	Fruit	[60]
138	3,4-dimethoxy-benzoic acid	Fruit	[58]
139	qrotocatecheuic acid	Fruit	[58]
140	engemol	Fruit	[58]
141	1,4-benzenediol	Fruit	[70]
142	1-ethoxy-4-methylbenzene	Fruit	[70]
143	1-methoxy benzaldehyde	Fruit	[70]
144	shikimic acid-3*-O-β-D*-mannopyranose (1″-6′)-*β-D*-mannopyranose	Fruit	[58]
145	iso-shikimic acid	Fruit	[58]
146	(3*R*,4*R*,6*S*)-3,4,6-trihydroxycyclohex-1-enecarboxylic acid	Fruit	[60]
147	shikimic acid	Fruit	[67]
148	shikimic acid methyl ester	Fruit	[58]
149	methyl shikimate	Fruit	[73]
150	4-methoxy cinnamic acid	Fruit	[60]
151	succinic acid	Fruit	[69,75,76]
152	9*-O-β-D*-glucopyranosyl-4-methoxy-cinnamic acid	Fruit	[73]
153	Magnolol	Fruit	[58]

**Table 5:** The terpenoids isolated from *Illicium verum*.

No.	Chemical Constituents	Parts	References
154	3,6-two hydroxy-1-menthene	End of distillation liquid	[85,86]
155	1*R*,3*R*,4*R*-trihydroxy-menthene	Fruit	[70]
156	3-hydroxy-4,5-methylenedioxyallyl-benzene	Root	[13]
157	2*α*,9*β*-dihydroxy-14 (10→1)-olivane-1(10)-ene	End of distillation liquid	[86]
158	naphthalenol, decahydro-2-methyl-5-methylene-8(1-methylethyl)	Fruit	[67]
159	1*α*-hydroxy-3-deoxypseudoanisatin	Fruit	[57]
160	(*E*)-1-[(3-methylbut-2-enyl)oxy]-2-methoxy-4-(prop-1-enyl) benzene	Root	[13]
161	4-allyl-2-(3-methylbut-2-enyl)-1,6-methylenedioxybenzene-3-ol	Root	[13]
162	illicinole	Root	[13]
163	(-)-illicinone-A	Root	[13]
164	4-allyl-4-(3-methylbut-2-enyl)-1,2-methylenedioxycyclohexa-2,6-dien-5-one	Root	[13]
165	veranisatin A	Fruit	[41]
166	veranisatin B	Fruit	[41]
167	veranisatin C	Fruit	[41]
168	anisatin	Fruit	[41]
169	neoanisatin	Fruit	[41]
170	vermiol N	Fruit	[67]
171	(3*R*,4*R*,3′*R*,4′*R*)-6,6′-dimethoxy-3,4,3′,4′-tetrahydro-2H,2′H- [3,3′] bichromenyl-4,4′-diol	Fruit	[67]
172	11-O-denbenzoyl-11*α*-O-2-methylcyclopent-1-enecarboxyltashironin	Root	[13]
173	tashironin	Root	[13]
174	ursolic acid	Root	[56,91]
175	schizandronic acid	Leaf	[91]
176	3,4-seco-(24Z)-cycloart-4(28),24-diene-3,26-dioic	Root, Leaf	[13]

**Table 6:** The other constituents isolated from *Illicium verum*.

No.	Chemical Constituents	Parts	References
177	(1′*R*,2′*R*)-anethole glycol	Fruit	[67]
178	(*S*)-2-amino-1-(4-methoxyphenyl) ethanol	Fruit	[68]
179	1-hexaco sanol	Root	[56]
180	4-methoxybenzenethanol	Fruit	[68]
181	4-methoxyphenol	Fruit	[68]
182	4-hydroxy-phenethylalcohol*-O-β-D*-glucopyranoside	Fruit	[72]
183	4-hydroxybenzaldehyde	Fruit	[68]
184	4-hydroxy-3-(3-methylbut-3-en-2-yl) benzaldehyde	Root	[56]
185	vanillin	Fruit	[67]
186	dibutyl terephthalate	Fruit	[58]
187	4-methoxy-3*-O-β-D*-glucopyranosyloxy benzoic acid methyl ester	Fruit	[59]
188	3-methoxyl-4*-O-β-D*-glucopyranosyloxy benzoic acid methyl ester	Fruit	[59]
189	4*-O-β-D*-glucopyranosyloxy benzoic acid methyl ester	Fruit	[59]
190	dibutyl phthalate	Fruit	[74]
191	bis(2-propyloctyl) phthalate	Fruit	[70]
192	bis(2-propylheptyl) phthalate	Fruit	[70]
193	bis(1-methylpentyl) ester	Fruit	[70]
194	(*E*)-4-methoxy-2-(1′-propen-1′-yl)-phenol-1-O-*α-L*-arabinofuranosyl-(1′′′→6′′)-*β-D*-glucopyranoside	Fruit	[73]
195	(*E*)-4-methoxy-2-(1′-propen-1′-yl)-phenol-1-*O-α-L*-rhamnopyranosyl-(1′′′→6′′)-*β-D*-glucopyranoside	Fruit	[73]
196	(*R*)-sec-butyl-*β-D*- glucopyranoside	Fruit	[65]
197	(*E*)-1,2-bis(4-methoxyphenyl) ethene	Fruit	[60]
198	thero-4-hydroxyphenylpropyan-7,8-diol 7-O-methyl ether	Fruit	[68]
199	*P*-hydroxyacetophenone	Fruit	[68]
200	fructose	Fruit	[58]
201	*β*-sitosterol	Root, Fruit	[56,70]

**Table 7:** The prescriptions of modern Chinese patent medicines (https://www.yaozh.com/, accessed on 15 October 2021).

Prescription Name	Main Composition	Clinical Uses	Prescription Sources
Jiebei Zhike Qutan Pian	*Platycodon grandiflorum.* (Jacq.) A. DC. (*225 g*)/*Polygala tenuifolia* Willd (44.12 g)/*Fritillaria cirrhosa* D. Don (150 g)/eucalyptus oil (2 g)/Star anise oil (2 g)/*Glycyrrhiza uralensis* Fisch. (25 g)/ammonium chloride (100 g)/crospovidone (10 g)/sodium starch glycolate (12 g)	Lung heat cleaning, cough-reducing and phlegm-reducing	Guo Jia Zhong Cheng Yao Biao Zhun Hui Bian (2002)
Beishen Pingchuan Jiaonang	*Herba Ardisiae* Japonicae (550 g)/*Ephedra sinica* Stapf (500 g)/*Illicium verum* Hook. F (180 g)/*Fritillaria thunbergii* Miq. (400 g)/*Pseudostellaria heterophylla* (Miq.) Pax ex Paxet Hoffm. (300 g)/*Siraitia grosuenorii*., (Swingle) C. Jeffreyex A. M. Lu et Z. Y. Zhang (265 g)/*Gekko gecko* Linnaeus (100 g)/*Glycyrrhiza uralensis* Fisch. (200 g)/starch (17 g)	Warming lungs to eliminate cold, anti-cough and anti-asthmatic	Guo Jia Zhong Cheng Yao Biao Zhun Hui Bian (2002)
Jiuji Xingjun Jiaonang	borneol (21.74 g)/*Scutellaria baicalensis* Georgi (21.41 g)/*Dryopteris crassirhizoma* Nakai (21.41 g)/*Mentha haplocalyx* Briq (27.34 g)/*Asarum heterotropoides* Fr. Schmidt (10.54 g)/*Angelica dahurica* (Fisch.ex Hoffm) Benth. et Hook (10.54 g)/*Gleditsia sinensis* Lam. (3.62 g)/*Atractylodes lancea* (Thunb.) DC. (10.54 g)/*Nardostachys jatamansi* DC (21.41 g)/theophylline (20.42 g)/realgar (6.92 g)/cinnabaris (8.23 g)/*Cinnamomum cassia* Presl (28.32 g)/*Alpinia officinarum* Hance (10.54 g)/*Illicium verum* Hook. F. (21.41 g)/*Eugenia caryophyllata* Thunb. (10.54 g)/*Aucklandia lappa* Decne. (21.41 g)/*Citrus reticulata* Blanco (21.41 g)/*Glycyrrhiza uralensis* Fisch. (32.38 g)	Eliminating stagnated food, relieving pain and diarrhea	Guo Jia Zhong Cheng Yao Biao Zhun Hui Bian (2002)
Jiuji Xingjun San	*Cinnamomum camphora* (L.) Presl (66 g)/*Scutellaria baicalensis* Georgi (64.5 g)/*Dryopteris crassirhizoma* Nakai (64.5 g)/*Mentha haplocalyx* Briq (82.5 g)/*Asarum heterotropoides* Fr. Schmidt (32.3 g)/*Angelica dahurica* (Fisch.ex Hoffm.) Benth. et Hook (32.3 g)/*Gleditsia sinensis* Lam. (11 g)/*Atractylodes lancea.* (Thunb.) DC. (32.3 g)/*Nardostachys jatamansi* DC. (64.5 g)/tea (62 g)/realgar (21 g)/cinnabaris (25 g)/*Cinnamomum cassia* Presl (86 g)/*Alpinia officinarum* Hance. (32.3 g)/*Illicium verum* Hook. F. (64.5 g)/*Eugenia caryophyllata* Thunb. (32.3 g)/*Aucklandia lappa* Decne. (64.5 g)/*Citrus reticulata* Blanco (64.5 g)/*Glycyrrhiza uralensis* Fisch. (98 g)	Eliminating stagnated food, relieving pain and diarrhea	Guo Jia Zhong Cheng Yao Biao Zhun Hui Bian (2002)
Xiao’er Kesouning Tangjiang	Tolu Bals (6 g)/Extractum Scillae. (2.4 g)/star anise oil (0.04 mL)/*Mentha haplocalyx* Briq. (0.15 g)/benzoic acid (1 g)/sucrose (510 g)/talc (6 g)	Expelling phlegm and arrest coughing	Guo Jia Zhong Cheng Yao Biao Zhun Hui Bian (2002)
Fufang Yatongning Chaji	*Pinus massoniana* Lamb. (120 g)/*Zanthoxylum schinifolium* Sieb. et Zucc. (90 g)/*Cinnamomum camphora* (L.) Presl. (22 g)/*Eugenia caryophyllata* Thunb. (15 g)/*Mentha haplocalyx* Briq (13 g)/*Schizonepeta tenuifolia* Briq. (10 g)/*Piper longum* L. (10 g)/*Artemisia scoparia* Waldst.et Kit. (10 g)/*Glycyrrhiza uralensis* Fisch. (10 g)/*Illicium verum* Hook. F. (10 g)	Detumescence and pain relief	Guo Jia Zhong Cheng Yao Biao Zhun Hui Bian (2002)
Shuwei Yaojiu	*Osmunda japonica* Thunb. (100 g)/*Amomum tsao-ko* Crevost et Lemaire. (10 g)/*Cinnamomum cassia* Presl (10 g)/*Glycyrrhiza uralensis* Fisch./*Illicium verum* Hook. F. (20 g)	Warming spleen and stomach for dispelling cold, regulating vital energy and alleviating pain	Guo Jia Zhong Cheng Yao Biao Zhun Hui Bian (2002)
Guixiang Qushu San	*Mentha haplocalyx* Briq (60 g)/*Cinnamomum camphora* (L.) Presl (100 g)/*Eugenia caryophyllata* Thunb. (7.5 g)/*Cinnamomum cassia* Presl (7.5 g)/*Illicium verum* Hook. F.(7 g)/*Glycyrrhiza uralensis* Fisch. (150 g)/Carmine (4.8 g)/Starch(520 g)/Dextrin(46 g)	Fragrant herbs repelling foulness, dispelling cold and relieving heat, relieving heat and providing energy	Guo Jia Zhong Cheng Yao Biao Zhun Hui Bian (2002)
LonghuRendan	*Mentha haplocalyx* Briq (40 g)/*Cinnamomum camphora* (L.) Presl (30 g)/*Eugenia caryophyllata* Thunb. (25 g)/*Amomum villosum* Lour. (25 g)/*Illicium verum* Hook. F. (15 g)/*Cinnamomum cassia* Presl (40 g)*, Piper nigrum L.* (15 g)/*Aucklandia lappa* Decne. (15 g)/*Zingiber officinale* Rosc. (25 g)/*Acacia catechu.* (L. f.) Willd. (200 g)/*Glycyrrhiza uralensis* Fisch. (364.1 g)/glutinous rice flour (180 g)/sodium benzoate (5 g)	Inducing resuscitation, eliminating turbid and summer heat, warming middle energizer to arrest vomiting	Guo Jia Zhong Cheng Yao Biao Zhun Hui Bian (2002)
Hantongle Refudai	*Aconitum carmichaelii* Debx. (120 g)/*Aconitum kusnezoffii* Reichb. (120 g)/*Ephedra sinica* Stapf. (120 g)/*Angelica sinensis* (Oliv.) Diels (247 g)/*Euodia rutaecarpa* (Juss.) Benth. (498 g)/*Atractylodes lancea* (Thunb.) DC. (247 g)/*Illicium verum* Hook. F. (80 g)/*Kaempferia galanga* Linn. (100 g)/*Mentha haplocalyx* Briq. (52 g)/Camphor (34 g)/*Cinnamomum camphora* (L.) Presl. (34 g)/methyl salicylate (51 g)/iron dust (31,106 g)/aluminum (552 g)/vermiculite (8818 g)/sodium chloride (1066 g)/cuprous chloride (10,066 g)	Relieving rheumatism and cold, relaxing the muscles and stimulating blood circulation	Guo Jia Zhong Cheng Yao Biao Zhun Hui Bian (2002)
Shexiang Zhuanggu Babugao	*Moschus berezovskii* Flerov. (0.63 g)/leopard bone (0.6 g)/*Aconitum carmichaelii* Debx. (158 g)/*Aconitum kusnezoffii* Reichb. (158 g)/*Ephedra sinica Stapf* (158 g)/*Angelica sinensis* (Oliv.) Diels (325 g)/*Kaempferia galanga* L. (132 g)/*Angelica dahurica* (Fisch.ex Hoffm.) Benth. et Hook. (200 g)/*Atractylodes lancea* (Thunb.) DC (325 g)/*Illicium verum* Hook. F. (105 g)/*Zingiber officinale* Rosc. (446 g)/*Cinnamomum camphora* (L.) Presl (80 g)/*Mentha haplocalyx* Briq (105 g)/castor oil (50 g)/chondroitin sulfate sodium (3 g)/methyl salicylate (67 g)/gelatin (80 g)/diphenhydramine hydrochloride (16 g)/sodium polyacrylate (273 g)/vinyl alcohol (50 g)/carboxymethylcellulose sodium (45 g)/gelatin (91 g)/kaolin (364 g)/glycerol (1365 g)/sorbitol (500 g)	Relieving rheumatism and cold, promoting blood circulation to relieve pain	Guo Jia Zhong Cheng Yao Biao Zhun Hui Bian (2002)
SijiYou	Pinus tabuliformis Carr. (10 g)/peppermint oil (430 g)/*Cinnamomum cassia* Presl (2.5 g)/star anise oil (17.5 g)/methyl salicylate (490 g)/camphor (15 g)/methyl salicylate (490 g)	Qufeng and exicting	Wei Sheng Bu Zhun Zhong Yao Cheng Fang Zhi Ji (1994)
Qixiang Zhitong Wan	*Vladimiria souliei* (Franch.) Ling (160 g)/*Aucklandia lappa* Decne. (20 g)/*Aquilaria sinensis* (Lour.) Gilg (20 g)/*Dalbergia odorifera* T. Chen (80 g)/*Foeniculum vulgare* Mill. (80 g)/*Illicium verum* Hook. F. (80 g)/*Eugenia caryophyllata* Thunb. (80 g)/*Boswellia carterii* Birdw. (80 g)/*Pogostemon cablin* (Blanco) Benth. (80 g)	Warming spleen and stomach for dispelling cold; promoting Qi circulation to stop pain	Wei Sheng Bu Zhun Zhong Yao Cheng Fang Zhi Ji (1990)
Fengshang Zhitong Gao	*Cinnamomum cassia* Presl (45 g)/*Illicium verum* Hook. F. (24 g)/*Eugenia caryophyllata* Thunb. (12 g)/*Xanthium sibiricum* Patr. (30 g)/*Cinnamomum japonicum* Sieb. (C.chekiangense Nakai) (17 g)/*Ricinus communis* L. (90 g)/*Nardostachys jatamansi* DC. (17 g)/*Arisaema erubescens* (Wall.) Schott (90 g)/*Angelica dahurica* (Fisch.ex Hoffm.) Benth. Et Hook. (30 g)/*Piper kadsura (Choisy)Ohwi.* (90 g)/*Rheum palmatum L*. (30 g)/*Pinellia ternate* (Thunb.) Breit. (90 g)/*Notopterygium incisum* Ting ex H. T. Chang (60 g)/*Aconitum carmichaelii* Debx. (90 g)/*Angelica pubescens* Maxim.f. biserrata Shan et Yuan (60 g)/*Aconitum kusnezoffii Reichb.* (90 g)/*Asarum heterotropoides Fr. Schmidt*. (30 g)/*Siegesbeckia orientalis L.* (90 g)/*Cinnamomum cassia Pres* (90 g)/*Angelica sinensis* (Oliv.) Diels (30 g)/*Ephedra sinica* Stapf. (90 g)	Stimulating the circulation of blood and causing the muscles and joints to relax, promoting the circulation of blood to relieve pain	Wei Sheng Bu Zhun Zhong Yao Cheng Fang Zhi Ji (1990)
Liuwei Wan	*Aconitum carmichaelii* Debx. (180 g)/*Aconitum kusnezoffii* Reichb. (180 g)/*Ephedra sinica* Stapf (180 g)/*Atractylodes lancea* (Thunb.) DC. (360 g)/*Angelica sinensis* (Oliv.) Diels (360 g)/*Angelica dahurica* (Fisch.ex Hoffm.) Benth. et Hook (229 g)/*Zingiber officinale* Rosc. (485 g)/*Kaempferia galanga* L. (146 g)/*Illicium verum* Hook. F. (114 g)/*Mentha haplocalyx* Briq (80 g)/*Cinnamomum camphora* (L.) Presl (60 g)/camphor (40 g)/methyl salicylate (60 g)	Nourishing the liver and kidneys	Wei Sheng Bu Zhun Zhong Yao Cheng Fang Zhi Ji (1990)
Shixiang Nuanqi Gao	*Illicium verum* Hook. F. (120 g)/*Foeniculum vulgare* Mill. (120 g)/*Lindera aggregate* (Sims)Kos-term. (120 g)/*Cyperus rotundus* L. (120 g)/*Angelica sinensis* (Oliv.) Diels (120 g)/*Angelica dahurica* (Fisch.ex Hoffm.) Benth. et Hook (120 g)/*Eugenia caryophyllata* Thunb. (30 g)/*Cinnamomum cassia* Presl (30 g)/*Aquilaria sinensis* (Lour.) Gilg (30 g)/*Boswellia carterii* Birdw. (30 g)/*Commiphora myrrha* Engl. (30 g)/*Aucklandia lappa* Decne. (30 g)	Warming the spleen and stomach for dispelling cold, stopping pain	Wei Sheng Bu Zhun Zhong Yao Cheng Fang Zhi Ji (1991)
Sanceng Huixiang Wan	*llicium verum* Hook. F. (200 g)/*Melia toosendan* Sieb. et Zucc. (200 g)/*Aucklandia lappa* Decne. (200 g)/*Poria cocos* (Schw.) Wolf (800 g)/*Glehnia littoralis* Fr. Schmidtex Miq. (200 g)/*Piperlongum* L. (200 g)/*Areca catechu* L. (100 g)/*Aconitum carmichaelii* Debx. (100 g)	Warming meridians to dissipate cold, promoting Qi circulation to stop pain	Wei Sheng Bu Zhun Zhong Yao Cheng Fang Zhi Ji (1991)
Bajiao Huixiang Shui	star anise oil (20 mL)/ethanol (570 mL)	Flavor correction, Qufeng	Wei Sheng Bu Zhun Zhong Yao Cheng Fang Zhi Ji (1993)
Shangshi qutong Gao	*Aconitum carmichaelii* Debx. (180 g)/*Aconitum kusnezoffii* Reichb.(180 g)/*Ephedra sinica* Stapf (180 g)/*Atractylodes lancea* (Thunb.) DC. (360 g)/A*ngelica sinensis* (Oliv.) Diels (360 g)/*Angelica dahurica* (Fisch. ex Hoffm.) Benth. et Hook (229 g)/*Zingiber officinale* Rosc. (485 g)/*Kaempferia galanga* L. (146 g)/*Illicium verum* Hook. F. (114 g)/*Mentha haplocalyx* Briq (80 g)/*Cinnamomum camphora* (L.) Presl (60 g)/camphor (40 g)/methyl salicylate (60 g)	Treating rheumatism, stopping pain	Wei Sheng Bu Zhun Zhong Yao Cheng Fang Zhi Ji (1993)
Suhe Wan	*Liquidambar orientalis* Mill. (10 g)/*Eugenia caryophyllata* Thunb. (120 g)/*Styrax tonkinensis* (Pierre) Craib ex Hart. (20 g)/*Boswellia carterii* Birdw. (10 g)/*Aucklandia lappa* Decne. (20 g)/*Santalum album* L. (20 g)/*Illicium verum* Hook. F./*Cyperus rotundus* L. (20 g)/*Atractylodes macrocephala* Koidz. (20 g)/*Terminalia chebula* Retz. (20 g)/*Piperlongum* L. (20 g)/cinnabaris (10 g)/*Cinnamomum camphora* (L.) Presl (10 g)	Dispelling the wind, analgesia, eliminating phlegm	Wei Sheng Bu Zhun Zhong Yao Cheng Fang Zhi Ji (1997)
Yulong You	*Zingiber officinale* Rosc. (48 g)/*Paeonia lactiflora* Pall. (48 g)/*Arisaema erubescens* (Wall.) Schott (16 g)/*Aconitum kusnezoffii* Reichb. (16 g)/*Aconitum carmichaelii* Debx. (16 g)/*Aconitum carmichaelii* Debx. (16 g)/*Boswellia carterii* Birdw. (10.7 g)/*Angelica dahurica* (Fisch.ex Hoffm.) Benth. et Hook (8 g)/*Clematis chinensis* Osbeck(8 g)/*Asarum heterotropoides* Fr. Schmidt (6.4 g)/*Rhus chinensis* Mill. (2.7 g)/Mentha haplocalyx Briq. (56 mL)/ambrum (36 g)/*Mentha haplocalyx* Briq (20 g)/*Ocimum gratissimum* L. (5.6 mL)/*Cinnamomum cassia* Presl (3.6 mL)/star anise oil (3.6 mL)/methyl salicylate (56 mL)/camphor (56 g)	Qufeng, removing blood stasis, relieving pain	Wei Sheng Bu Zhun Zhong Yao Cheng Fang Zhi Ji (1997)
Chanling Wan	*Panax ginseng* C. A. Mey. (90 g)/*Atractylodes macrocephala* Koidz. (15 g)/*Angelica sinensis* (Oliv.) Diels (15 g)/*Ligusticum chuanxiong* Hort. (90 g)/*Atractylodes lancea* (Thunb.) DC. (240 g)/*Polygonum multiflorum* Thunb. (15 g)/*Schizonepeta tenuisfolia* Briq. (90 g)/*Saposhnikovia divaricate* (Turcz) Schischk. (90 g)/*Ephedra sinica* Stapf (90 g)/*Aconitum carmichaelii* Debx. (90 g)/*Aconitum kusnezoffii Reichb*. (90 g)/*Angelica dahurica* (Fisch.ex Hoffm.) Benth. et Hook (90 g)/*Asarum heterotropoides* Fr. Schmidt. (15 g)/*Illicium verum* Hook. F. (90 g)/*Aucklandia lappa* Decne. (15 g)/*Anemone raddeana* Regel (15 g)/*Platycodon grandiflorum* (Jacq.) A. DC. (90 g)/*Daemonorops draco* Bl. (15 g)/*Glycyrrhiza uralensis* Fisch. (60 g)	Reinforcing Qi and nourishing blood, dissipating the wind and relieving pain	Wei Sheng Bu Zhun Zhong Yao Cheng Fang Zhi Ji (1995)
Hupo Zhitong Gao	*Kaempferia galanga* L (140 g)/*Acorus tatarinowii* Schott (70 g)/*Coptis chinensis* Franch. (42 g)/*Strychnos nux-vomica* L. (140)/*Mylabris phalerata* Pallas (2.8 g)/*Clematis chinensis* Osbeck (280 g)/*Arisaema erubescens* (Wall.) Schott (105 g)/*Bufo bufo gargarizans* Cantor (5.7 g)/*Ambrum* (20.6 g)/*Ocimum gratissimum L.* (9.8 g)/*Mentha haplocalyx* Briq (22 g)/star anise oil (14.7 g)/*Cinnamomum tamala* (Bauch.-Ham.) Nees et Eberm (7.4 g)/*Cinnamomum camphora* (L.) Presl (14.7 g)/camphor (14.7 g)	Blood activating and phlegm resolving, eliminating the mass and relieving swelling, dredging collaterals and relieving pain	Wei Sheng Bu Zhun Zhong Yao Cheng Fang Zhi Ji
Ruihua You	*Mentha haplocalyx* Briq (320 g)/methyl salicylate (230 g)/star anise oil (10 g)/camphor (70 g)/*Eucalyptus globulus* Labill. (70 g)	Qufeng and relieving pain, relieving itching	Wei Sheng Bu Zhun Zhong Yao Cheng Fang Zhi Ji (1997)
Nuanqi Gao	*Angelica sinensis* (Oliv.) Diels (80 g)/*Angelica dahurica* (Fisch.ex Hoffm.) Benth. et Hook (80 g)/*Lindera aggregate* (Sims) Kos-term. (80 g)/*Foeniculum vulgare* Mill. (80 g)/*Illicium verum* Hook. F. (80 g)/*Aucklandia lappa* Decne. (40 g)/*Cyperus rotundus* L. (80 g)/*Boswellia carterii* Birdw. (20 g)/*Eugenia caryophyllata* Thunb. (20 g)/*Commiphora myrrha* Engl. (20 g)/*Cinnamomum cassia* Presl (20 g)/Aquilaria sinensis (Lour.) Gilg (20 g)/*Moschus berezovskii* Flerov. (3 g)	Dispelling cold, promoting Qi circulation to stop pain	Wei Sheng Bu Zhun Zhong Yao Cheng Fang Zhi Ji (1998)
Yaoshen Gao	*Cistanche deserticola* Y.C. Ma/*Illicium verum* Hook. F./*Rehmanniae radix praeparata*/*Psoralea corylifolia* L./*Epimedium brevicornu* Maxim./*Cnidium monnieri* (L.) Cuss./*Achyranthes bidentata* Bl./*Dipsacus asper* Wall. ex Henry/*Glycyrrhiza uralensis* Fisch./*Eucommia ulmoides* Oliv./*Cuscuta australis* R.Br./*Lycium barbarum* L./*Plantago asiatica* L./*Foeniculum vulgare* Mill./*Aconitum carmichaelii* Debx./*Schisandra chinensis* (Turcz.) Baill./*Boswellia carterii* Birdw./*Commiphora myrrha* Engl./*Eugenia caryophyllata* Thunb./*Cynomorium songaricum* Rupr./Camphor/*Cinnamomum camphora* (L.) Presl/*Mentha haplocalyx* Briq/*Cinnamomum cassia* Presl/methyl salicylate/*Liquidambar formosana* Hance/diphenhydramine hydrochloride	Warming kidneys and enhancing Yang, strengthening tendons and bones, Qufeng and relieving pain	Wei Sheng Bu Zhun Zhong Yao Cheng Fang Zhi Ji (1998)
Huixiang Juhe Wan	*Oeniculum vulgare* Mill. (40 g)/*Illicium verum* Hook. F. (40 g)/*Citrus reticulata* Blanco (40 g)/*Litchi chinensis* Sonn. (80 g)/*Psoralea corylifolia* L. (20 g)/*Cinnamomum cassia* Presl (16 g)/*Melia toosendan* Sieb. et Zucc. (80 g)/*Corydalis yanhusuo* W.T. Wang (40 g)/*Curcuma phaeocaulis* VaL (20 g)/*Aucklandia lappa* Decne. (20 g)/*Cyperus rotundus* L. (40 g)/*Citrus reticulata* Blanco (40 g)/*Laminariajaponica* Aresch. (40 g)/*Areca catechu* L. (40 g)/*Boswellia carterii* Birdw. (20 g)/*Prunus persica* (L.) Batsch (16 g)/*Manis* (20 g)	Dispersing cold, conducting Qi, detumescence and pain relief	Pharmacopoeia of the People’s Republic of China (2020)
Qianlietong Pian	Receptaculum Fici Pumilae (400 g)/*Astragalus membranaceus* (Fisch.) Bge. var. mongholicus (Bge.) Hsiao (464 g)/*Plantago asiatica* L. (264 g)/*phellodendron amurense* Rupr. (336 g)/*Anemone raddeana* Regel (336 g)/*Taraxacum mongolicum* Hand. -Mazz. (336 g)/*Lycopus lucidus* Turcz. var. hirtus Regel (336 g)/*Ambrum* (75 g)/star anise oil (1.7 mL)/cinnamon oil (0.88 mL)	Clearing heat and promoting diuresis, transforming stasis and dissipating binds	Pharmacopoeia of the People’s Republic of China (2020)
Suoyang Gujing Wan	*Cynomorium songaricum* Rupr. (20 g)/Cistan*che deserticola* Y.C. Ma (25 g)/*Morinda officinalis* How (30 g)/*Psoralea corylifolia* L. (25 g)/*Cuscuta australis* R. Br. (20 g)/*Eucommia ulmoides* Oliv. (25 g)/*Illicium verum* Hook. F. (25 g)/*Allium tuberosum* Rott L. ex Spreng. (20 g)/*Euryale ferox* Salisb. (20 g)/*Nelumbo nucifera* Gaertn. (20 g)/*Nelumbo nucifera* Gaertn. (25 g)/*Ostrea gigas* Thunberg (20 g)/dragonsbones (20 g)/*Cornu Cervi* Degelatinatum (2 g)/*Rehmanniaglutinosa* (Gaetn.) Libosch. ex Fisch. et Mey. (56 g)/*Cornus officinalis* Sieb. et Zucc. (17 g)/*Paeonia suffruticosa* Andr. (11 g)/*Dioscorea opposita* Thunb. (56 g)/*Poria cocos* (Schw.) Wolf (11 g)/*Alisma orientale* (Sam.) Juzep. (11 g)/*Anemarrhena asphodeloides* Bge. (4 g)/*Phellodendron chinense* Schneid. (4 g)/*Achyranthes bidentata* Bl. (20 g)/haltum (25 g)	Warming the kidneys and stopping emission	Pharmacopoeia of the People’s Republic of China (2020)
Nuanqi Gao	*Angelica sinensis* (Oliv.) Diels (80 g)/*Angelica dahurica* (Fisch.ex Hoffm.) Benth. et Hook (80 g)/*Lindera aggregate* (Sims) Kos-term. (80 g)/*Foeniculum vulgare* Mill. (80 g)/*Illicium verum* Hook. F. (80 g)/*Aucklandia lappa* Decne. (40 g)/*Cyperus rotundus* L. (80 g)/*Boswellia carterii* Birdw. (20 g)/*Eugenia caryophyllata* Thunb. (220 g)/*Commiphora myrrha* Engl. (20 g)/*Cinnamomum cassia* Presl (20 g)/*quilaria sinensis* (Lour.) Gilg (20 g)/*Moschus berezovskii* Flerov (3 g)	Dispelling cold, promoting Qi circulation to stop pain	Pharmacopoeia of the People’s Republic of China (2020)
Qianlietong Shuan	*Ficus pumila* Linn./*Astragalus membranaceus* (Fisch.) Bge.var.mongholicus (Bge.) Hsiao/*Plantago asiatica* L./*Phellodendron chinense* Schneid./*Anemone raddeana* Regel/*Taraxacum mongolicum* Hand. -Mazz./*Lycopus lucidus* Turcz. var. hirtus Regel/star anise oil/cinnamon oil/ambrum	Clearing away the heat-evil, expelling superficial evils, clearing heat and promoting diuresis, promoting the circulation of Qi and blood, anti-inflammatory and analgesic effects, dispelling stasis and freeing strangury	Xin Yao Zhuan Zheng Biao Zhun (2002)
SiJi PingAn You	*Mentha haplocalyx* Briq/*Pogostemon cablin* (Blanco) Benth./*Citrus reticulata* Blanco/*Mentha haplocalyx* Briq./camphor/*Cinnamomum cassia* Presl/*Glycyrrhiza uralensis* Fisch./*Gardenia jasminoides* Ellis/*Cinnamomum camphora* (L.) Presl/Borneo/*Angelica sinensis* (Oliv.) Diels/*Illicium verum* Hook. F./*Ligusticum chuanxiong* Hort./*Eugenia caryophyllata* Thunb./clove oil/*Aucklandia lappa* Decne./*Cinnamomum cassia* Presl/*Daemonorops draco* Bl.	Dispelling cold and relieving the heat	Xin Yao Zhuan Zheng Biao Zhun (2002)
Jiuxiang Zhitong Wan	*Vladimiria souliei* (Franch.) Ling (160 g)/*Aucklandia lappa* Decne. (20 g)/*Aquilaria sinensis* (Lour.) Gilg (20 g)/*Dalbergia odorifera* T. Chen (80 g)/*Foeniculum vulgare* Mill. (80 g)/*Illicium verum* Hook. F. (80 g)/*Eugenia caryophyllata* Thunb. (80 g)/*Boswellia carterii* Birdw. (80 g)/*Pogostemon cablin* (Blanco) Benth. (80 g)	Dispelling cold, promoting Qi circulation to stop pain	Pharmacopoeia of the People’s Republic of China (2015)
Mentha Laryngitic Tablets	caramel/star anise oil/*Eucalyptus globulus* Labill./chlorobutanol/sodium benzoate/*Mentha haplocalyx* Briq	Algefacient, antiseptic, antalgic	Pharmacopoeia of the People’s Republic of China (1963)
Ganlanjing Keli	*Canarium album* Raeusch./*Pinelliae rhizome praeparatum cum* zingibere et al. umine/*Perilla frutescens* (L.) Britt./*Prunus armeniaca* L.var.ansu Maxim./*Alpinia officinarum* Hance/*Crataegus pinnatifida* Bge. var. major N. E. Br./*Foeniculum vulgare* Mill./*Zanthoxylum schinifolium* Sieb. et Zucc./*Poncirus trifoliata* (Linn.) Raf./*Glycyrrhiza uralensis* Fisch./*Citrus reticulata* Blanco/*Cyperus rotundus L*./*Magnolia officinalis* Rehd. et Wils./*Mentha haplocalyx* Briq/*Amomum villosum* Lour./*Cinnamomum tamala* (Bauch-Ham.) Nees et Eberm/*Eugenia caryophyllata* Thunb./*Illicium verum* Hook. F.	Appetizing, promoting digestion to eliminate stagnation, driving away summer heat, antidiarrheic, giving appetite	Xin Bian Guo Jia Zhong Cheng Yao (2002)
Hantong leYunji	*Aconitum carmichaelii* Debx. (2.2 mg)/*Aconitum kusnezoffii* Reichb. (2.2 g)/*Ephedra sinica* Stapf. (2.2 g)/*Angelica sinensis* (Oliv.) Diels (4.5 g)/*Euodia rutaecarpa* (Juss.) Benth. (9.1 mL)/*Atractylodes lancea* (Thunb.) DC. (4.5 mg)/*Illicium verum* Hook. F. (1.5 mg)/*Kaempferia galanga* Linn. (1.8 mg)/*Mentha haplocalyx* Briq (0.95 mg)/camphor (0.62 mg)/*Cinnamomum camphora* (L.) Presl (0.62 mg)/methyl salicylate (0.93 mL/g)	Expelling wind and clearing away cold, relaxing the muscles and stimulating blood circulation	Xin Bian Guo Jia Zhong Cheng Yao (2002)
Jiuxiang Zhitong Wan	*Vladimiria souliei* (Franch.) Ling (160 g)/*Eugenia caryophyllata* Thunb. (20 g)/*Aquilaria sinensis* (Lour.) Gilg (20 g)/*Dalbergia odorifera* T. Chen (80 g)/*Foeniculum vulgare* Mill. (80 g)/*Illicium verum* Hook. F. (80 g)/*Eugenia caryophyllata* Thunb (80 g)/*Boswellia carterii* Birdw. (80 g)/*Pogostemon cablin* (Blanco) Benth. (80 g)	Dispelling cold, promoting Qi circulation to stop pain	Pharmacopoeia of the People’s Republic of China (2020)

**Table 8:** Prescriptions of traditional Chinese patent medicine.

Prescription Name	Main Composition	Traditional Uses	Prescription Sources
Huixiang Wan	*Illicium verum* Hook. F. (25 g)/*Melia toosendan* Sieb.et Zucc. (5 g)/*Angelica pubescens* Maxim.f. biserrata Shan et Yuan (25 g)/*Glycyrrhiza uralensis* Fisch. (25 g)/*Eriocaulon buergerianum* Koern. (25 g)/matcha (50 g)/*Atractylodes lancea* (Thunb.) DC (50 g)	Tetanus	Pu Ji Fang (AD1390)
Huixiang Cangzhu Wan	*Atractylodes lancea* (Thunb.) DC. (500 g)/*Psoralea corylifolia* L. (100 g)/*Illicium verum* Hook. F. (100 g)/*Morinda officinalis* How (100 g)/*pomoea cairica* (Linn.) Sweet (100 g)	Swelling and prolapse of one of the scrota, twinge	Pu Ji Fang (AD1390)
Chenxiang Jiji Wan	*Citrus aurantium* L. (150 g)/*Melia toosendan* Sieb. et Zucc. (150 g)/*Morinda officinalis* How (150 g)/*Allium tuberosum* Rottl. ex Spreng (150 g)/*Illicium verum* Hook. F. (150 g)/*Poria cocos* (Fr.) Wolf. (150 g)/*Aucklandia lappa* Decne. (50 g)/*Aquilaria sinensis* (Lour) Gilg (50 g)/*Moschus berezovskii* Flerov (100 g)/halitum (50 g)/*Equus caballus orientalis* Noack (1 g)	Reglution of Qi, afrodyn	Pu Ji Fang (AD1390)
Baishai Dan	*Illicium verum* Hook. F. (100 g)/*Aconitum carmichaelii* Debx. (100 g)/*Rhizoma Areactylodis Lanceae* (100 g)/*Rehmannia glutinosa* (Gdertn) Iibosch. ex Fisch. et Mey. (150 g)/*Poria cocos* (Fr.) Wolf. (100 g)/*Dioscorea opposita* Thunb. (100 g)	Enriching muscles, bones, arteries and veins, breaking obscure complexion, resolving visceral accumulation, reconciling viscera, getting rid of heart Qi, night sweat and wind disease, blacken hair and beard	Qi Fang Lei Bian (AD1644~1911)
Chenxiang Wan	*Aquilaria sinensis* (Lour.) Gilg (15 g)/*Aucklandia lappa* Decne. (15 g)/*Symplocos paniculata* (Thunb.) Miq. (15 g)/*Juglans regia* L. (15 g)/*Eugenia caryophyllata* Thunb. (15 g)/*Lycium barbarum* L. (15 g)/*Illicium verum* Hook. F. (15 g)/*Buthus martensii* Karsch (25 g)/*Foeniculum vulgare* Mill. (25 g)/*Melia toosendan* Sieb. et Zucc. (25 g)/*Trigonella foenum-graecum L.* (25 g)/*Psoralea corylifolia* L. (25 g)/*Manis* (15 g)/*Cuscuta australis* R. Br. (25 g)/*Dipsacus fullonum* L. (25 g)/*Polygala tenuifolia* Willd. (25 g)/*Allium tuberosum* Rottl. ex Spreng (25 g)/*Nelumbo nucifera* Gaertn. (15 g)/*Morinda officinalis* How (25 g)/*Dioscorea opposita* Thunb. (25 g)/*Cornus officinalis* Sieb. et Zucc. (25 g)/*Dioscorea opposita* Thunb. (25 g)/*Epimedium sagittatum* (Sieb. et Zucc.) Maxim. (10 g)/*Citrus reticulata* Blanco (15 g)/*Citrus reticulata* Blanco (15 g)/*Poria cocos* (Fr.) Wolf. (25 g)	Enhancing memory ability, reinforcing the five viscera, enriching muscles and bones, improving eyesight, consolidating the teeth	Yi Fang Lei Ju (AD1443~1447)
Da Huixiang Wan	*Crataegus pinnatifida* Bge. var. major N. E. Br. (200 g)/*Citrus reticulata* Blanco (100 g)/*Foeniculum vulgare* Mill. (100 g)/*Gardenia jasminoides* Ellis (100 g)/*Bupleurum chinense DC.* (50 g)/*Paeonia suffruticosa* Andr. (50 g)/*Prunus persica* (L.) Batsch (50 g)/*Illicium verum* Hook. F. (50 g)/*Euodia rutaecarpa* (Juss.) Benth. (25 g)	Head pain	XingYuan (AD1586)
Erxiang Wuzi Sanzhu Wan	*Illicium verum* Hook. F. (25 g)/*Inula helenium L.* (25 g)/*Areca catechu L*. (50 g)/*Melia toosendan* Sieb. et Zucc. (150 g)/*Cyperus rotundus* L. (50 g)/*Raphanus sativus* L. (100 g)/*Ipomoea cairica* (Linn.) Sweet (150 g)/*Euodia rutaecarpa* (Juss.) Benth. (150 g)/*Zanthoxylum ailanthoides* (150 g)/*Cornus officinalis* Sieb. et Zucc. (150 g)	Lumbago	Yong Lei Qian Fang (AD1331)
Guyuan Wan	*Illicium verum* Hook. F. (30 g)/*Rubus chingii* Hu (30 g)/*Foeniculum vulgare* Mill. (30 g)/*Poria cocos* (Fr.) Wolf. (30 g)/*Cyathula officinalis Kuan* (30 g)/Magnetitum (30 g)/*Apatite* (30 g)/*Psoralea corylifolia*L. (30 g)/*Aconitum carmichaeli* Debx.(30 g)/*Schisandra chinensis* (Turcz.) Baill. (60 g)/*Cuscuta australis* R. Br. (60 g)/*Cervus nippon* Temminck (75 g)/*Cistanche salsa* (C. A. Mey.) G. Beck (75 g)/*Plantago asiatica* L. (15 g)/*Moschus berezovskii* Flerov (1.5 g)/stalactitum (45 g)/*Aconitum carmichaelii* Debx. (45 g)/*Aconitum carmichaelii* Debx. (45 g)/*Cinnamomum cassia* Presl (45 g)/*Morinda officinalis* How (45 g)	Deficiency/weakness of primordial Qi	Zhu Shi Ji Yan Fang (AD1127~1279)
Huichun Wan	*Poria coco s* (Schw.) Wolf (30 g)/*Atractylodes macrocephala* Koidz. (30 g)/*Crataegus pinnatifida* Bge. var. major N. E. Br. (30 g)/*Citrus aurantium* L. (24 g)/*Illicium verum* Hook. F. (30 g)/*Cornus* (30 g)/*Citrus reticulata* Blanco (90 g)/*Litchi chinensis* Sonn. (30 g)	Hernia	She Sheng Zhong Miao Fang (AD1550)
Huixiang Jupi Jiu	*Illicium verum* Hook. F. (50 g)/*Citrus reticulata* Blanco (100 g)/*Amomum kravanh* Pierre ex Gagnep. (25 g)	Lower abdominal pain, chest and abdominal pain	Yi Tong (AD1556)
Huixiang San	*Aucklandia costus* Falc. (50 g)/*Camellia sinensis* (L.) O. Ktze. (50 g)/*Illicium verum* Hook. F. (25 g)/*Boswellia carterii* Birdw. (25 g)/*Panax ginseng* C. A. Mey. (25 g)/*Melia toosendan* Sieb. et Zucc. (125 g)/*Glycyrrhiza uralensis* Fisch. (75 g)/*Anemarrhena asphodeloides* Bge. (75 g)/*Foeniculum vulgare* Mill. (75 g)/*Fritillaria spp*. (75 g)/*Aquilaria sinensis* (Lour.) Gilg (10 g)/*Styrax tonkinensis* (Pierre) Craib ex Hart. (12.5 g)	Toothache	Yi Fang Lei Ju (AD1443~1447)
Huixiang Yin	*Illicium verum* Hook. F./*Cissusrepens* Lamk.	Hernia	Zhu Shi Ji Yan Fang (AD1127~1279)
Wuxian Zhushen Dan	*Illicium verum* Hook. F. (2.4 g)/*Psoralea corylifolia* L. (2.4 g)/*Eucommia ulmoides* Oliv. (2.4 g)/halitum (2.4 g)/*Cistanche deserticola* Y.C. Ma	Lumbago	Fu Shou Jing Fang (AD1530)

**Table 9:** A comparison of the common extraction methods used for obtaining star anise extracts.

Extraction Methods	Optimal Extraction Conditions	Extraction Rates (Retain Two Significant Digits)	Advantages	Disadvantages	References
Steam distillation (SD)	fruit size: 425 μm granulometry < 0.425 mm 8% mass 1 h water volume of 200 mL	10%	Simple equipment, convenient operation and low cost	Time-consuming and low oil yield	[99]
Ultrasonic-assisted extraction (UE)	solid-to-liquid ratio of 1:15 (g/mL) crushing particle size of 60 mesh ultrasonic time of 30 min	28%	Stable temperature, simple operation, short extraction time	Difficult and costly equipment manufacture	[100]
Supercritical fluid CO_2_ extraction (SCFE)	1 h extraction temperature 40 °C extraction pressure 250 bar	15%	Low temperature extraction No solvent residue	Strict requirements for equipment and materials	[9]
Microwave-assisted Soxhlet extraction (MAEE)	ratio of solvent to sample 17 mL/g extraction time 16 min microwave power 505 W	25%	Safe and environmentally friendly High extraction rate Short time	High operational requirements	[35,98,99]

## Data Availability

Not applicable.
